# Remodeling of leaf lipidome during cold acclimation, de-acclimation, and re-acclimation influences frost tolerance of *Festuca arundinacea*

**DOI:** 10.1093/pcp/pcag073

**Published:** 2026-05-29

**Authors:** Julia Górna, Dawid Perlikowski, Saleh Alseekh, Marcin Rapacz, Alisdair R Fernie, Magdalena Wójcik-Jagła, Arkadiusz Kosmala

**Affiliations:** Institute of Plant Genetics, Polish Academy of Sciences, Plant Physiology Team, Strzeszynska 34, 60-479, Poznan, Poland; Institute of Plant Genetics, Polish Academy of Sciences, Plant Physiology Team, Strzeszynska 34, 60-479, Poznan, Poland; Max Planck Institute of Molecular Plant Physiology, Department Gutjahr, Am Mühlenberg, 114476, Potsdam-Golm, Germany; Center of Plant Systems Biology and Biotechnology, Crop Quantitative Genetics, 14 St. Knyaz Boris I – Pokrastitel Str., 4023, Plovdiv, Bulgaria; University of Agriculture in Krakow, Faculty of Agriculture and Economics, Aleja Mickiewicza 21, 31-120, Krakow, Poland; Max Planck Institute of Molecular Plant Physiology, Department Gutjahr, Am Mühlenberg, 114476, Potsdam-Golm, Germany; University of Agriculture in Krakow, Faculty of Agriculture and Economics, Aleja Mickiewicza 21, 31-120, Krakow, Poland; Institute of Plant Genetics, Polish Academy of Sciences, Plant Physiology Team, Strzeszynska 34, 60-479, Poznan, Poland

**Keywords:** cold acclimation, de-acclimation, re-acclimation, frost tolerance, *Festuca arundinacea*, lipidome

## Abstract

Seasonal temperature increases in autumn and winter can significantly disrupt frost tolerance in plants. This inducible trait is acquired during cold acclimation (CA), reduced or lost during de-acclimation (DA), and potentially restored during re-acclimation (RA). The functionality of cellular membranes, closely associated with lipid composition, plays a crucial adaptive role in enabling plants to withstand low-temperature stress. However, studies focusing on effects of DA and RA on frost tolerance, particularly in forage grasses, remain limited. Here, we assessed frost tolerance in high frost tolerant (HFT) and low frost tolerant (LFT) genotypes of *Festuca arundinacea* following CA, DA, and RA, based on the assessment of their cellular membrane integrity as reflected by the parameter describing the temperature causing a 50% level of electrolyte leakage from cells. Analyses of membrane integrity and lipid composition during CA revealed that the HFT genotype maintained greater membrane stability through extensive remodeling of glycerolipids, including increased lipid unsaturation, particularly in phosphatidylethanolamine, preserved a balanced monogalactosyldiacylglycerol/digalactosyldiacylglycerol ratio, and enhanced triacylglycerol (TAG) accumulation, compared to the LFT genotype. DA reversed rearrangements in structural lipids and reduced frost tolerance, whereas RA induced strong genotype-dependent responses, especially in phosphoglycerolipid profiles. Analysis of mRNA accumulation profiles indicated key enzymes potentially involved in lipid remodeling. Major transcriptional changes were observed in phytyl ester synthase 1 (*PES1*), digalactosyldiacylglycerol synthase 1 (*DGD1*), and lipoxygenase family genes, indicating their importance during CA, DA, and RA. Collectively, these findings demonstrate, for the first time, that genotype-dependent lipid remodeling underlies the capacity of *F. arundinacea* to maintain and recover frost tolerance. We further suggest that stress-induced TAG accumulation may function as a protective metabolic buffer, sequestering excess free fatty acids, limiting lipid oxidation, and supporting membrane stability under low-temperature stress.

## Introduction

Winter hardiness refers to a complex set of adaptive molecular, physiological, and morphological traits that enhance the ability of plants to survive adverse environmental conditions during the winter season (Rapacz et al. 2014, Augustyniak et al. 2020). The main component of winter hardiness is frost tolerance, which is a polygenic trait affecting multiple physiological and metabolic processes that enhance plant cell survivability under 0°C temperatures (Marcellos and Single 1976). This trait is induced by cold acclimation (CA) (hardening), the process that requires plants’ exposure to low but not freezing temperatures. CA involves a series of biochemical and physiological changes. This includes cellular membranes remodeling (including modifications in membrane lipid composition) and activation of the antioxidant system to mitigate the damaging effects of reactive oxygen species (ROS) such as hydrogen peroxide (H₂O₂). Excess ROS levels have a cytotoxic effect, leading to lipid peroxidation, production of highly toxic malondialdehyde (MDA), protein denaturation, and DNA damage (Gill and Tuteja 2010, Choudhury et al. 2017, Augustyniak et al. 2018, Dreyer and Dietz 2018, Augustyniak et al. 2020, Devireddy et al. 2021, Asif et al. 2025). Low temperature also impairs photosynthetic electron transport. As such, the capacity of plants to acclimate their photosynthetic apparatus to low temperature is regarded as a key determinant of freezing tolerance and winter survival (Qian et al. 2024). In meadow fescue (*Festuca pratensis*), low-temperature waterlogging-induced changes in water-soluble carbohydrates and Rubisco activity were shown to modify chlorophyll fluorescence traits and thus the efficiency of photosynthetic acclimation to cold (Jurczyk et al. 2016). However, photosynthetic acclimation to cold depends not only on changes in proteins and soluble metabolites but also on remodeling of membrane lipids, since the degree of fatty-acid unsaturation and the relative abundance of chloroplast galactoglycerolipids are central for maintaining membrane fluidity and the proper function of PSI and photosystem II (PSII) at low temperature (Li and Yu 2018).

In the face of progressive climate change, the growing instability of temperature conditions in particular seasons poses new adaptation challenges for plants. Disruption of the seasonal thermal rhythm, particularly as a result of increasingly frequent episodes of warming in late autumn, winter, or early spring, result in de-acclimation (DA) (Kosová et al. 2025), significantly affecting the physiological processes responsible for the adaptation of plants to survive adverse winter conditions. DA can refer to the partial or complete loss of previously acquired frost tolerance by reversal of adaptive modifications, including a decrease of activity of antioxidant systems or a change in the composition and properties of membrane lipids. As a consequence, plants become more susceptible to frost damage, especially in the case of a sudden drop in temperature after a warmer period (Rapacz 1999, Kalberer et al. 2006, Rapacz et al. 2022, Stachurska et al. 2024, Kosová et al. 2025). Following the further lowering of temperatures, plants may restore the loss of frost tolerance in a re-acclimation (RA) process (Byun et al. 2014). Despite considerable research efforts, the molecular and physiological mechanisms underlying the sequence of CA, DA, and RA are still not fully understood (Kalberer et al. 2006, Rapacz et al. 2022, Vaitkevičiūtė et al. 2024).

Studies involving the complete plant CA—DA—RA sequence within a single, continuous experimental framework have been rarely reported (Byun et al. 2014). Most investigations, especially in monocots, have focused on individual phases of plant responses to fluctuating temperatures, frequently restricting analyses to the CA or DA stage (Kosmala et al. 2009, Augustyniak et al. 2018, Augustyniak et al. 2020, Wójcik-Jagła and Rapacz 2023). RA has been, nevertheless, only sporadically addressed, despite its potentially significant role in maintaining membrane integrity and tissue functionality during recurrent temperature fluctuations in winters (Vyse et al. 2019, Vaitkevičiūtė et al. 2022). Furthermore, the studies on the full CA, DA, RA sequence may be particularly relevant to understanding mechanisms of winter survival under climate change scenarios, where intermittent mid-winter warm spells could be associated with the disruption of adaptive processes crucial to survive low-temperature conditions, including frost during winter period (Kosová et al. 2025).

Cellular membranes are fundamental determinants of plant survival under environmental stresses, acting as both the first line of defense and the central interface for sensing and transducing stress-induced challenges (Stachurska et al. 2025). The plasma membrane, positioned at the interface between the cell and its surroundings, functions as a selective barrier that regulates the transport of ions and metabolites while coordinating signal perception and downstream adaptive responses. Its structural integrity and capacity to maintain homeostasis are essential for sustaining cellular functionality when plants face adverse conditions (Barrero-Sicilia et al. 2017, Maryam et al. 2025, Su et al. 2025). Environmental challenges, such as low temperature, impose severe structural and functional constraints on cellular membranes. These stresses disrupt lipid organization, alter membrane fluidity and flexibility, and modify protein–lipid interactions, compromising membrane integrity (Uemura et al. 1995, Thomashow 1999, Hatsugai and Katagiri 2018, Jahed et al. 2023, Maryam et al. 2025). Damage is not only confined to the plasma membrane since organellar membranes, including chloroplast envelopes and thylakoid membranes, are similarly affected, with direct consequences for photosynthetic performance and overall metabolic efficiency (Steponkus 1984, Degenkolbe et al. 2012). Plants, however, attenuate such effects through a dynamic remodeling of membrane lipids (Campos et al. 2003, Degenkolbe et al. 2012, Jahed et al. 2023).

Structural glycerolipids constitute the primary components of plant cellular membranes and can be divided into two groups: phosphoglycerolipids and galactoglycerolipids (Moellering and Benning 2011, Zhao et al. 2024). Phosphatidylcholine (PC), the main building block of extra-chloroplast membranes, primarily stabilizes the membrane and maintains structural integrity, while phosphatidylethanolamine (PE), due to its non-bilayer-forming, cone-shaped structure, enhances membrane flexibility, allowing dynamic remodeling under stress conditions (Yu et al. 2021, Zhao et al. 2024). In plants, PE is synthesized mainly via the cytidine diphosphate (CDP)-ethanolamine branch of the Kennedy pathway, whereas PC can be produced either from diacylglycerol (DAG) through the CDP-choline pathway or via sequential methylation of PE (Bates et al. 2013). Plastid lipids are mainly galactoglycerolipids, predominantly monogalactosyldiacylglycerol (MGDG), alongside digalactosyldiacylglycerol (DGDG). Collectively, these galactoglycerolipids constitute approximately 80% of plastid lipid content, playing a crucial role in the functionality of plastids, especially in the process of photosynthesis (Mackender and Leech 1974). MGDG, due to its non-bilayer-forming, cone-shaped structure, increases the fluidity of thylakoid membranes and the optimal organization of photosynthetic protein complexes. In contrast, DGDG, as a bilayer-forming galactoglycerolipid, stabilizes the bilayer regions of plastid membranes, maintaining membrane integrity and facilitating proper lipid–protein interactions (Rys et al. 2024, Zhao et al. 2024). MGDG in plastids is synthesized by monogalactosyldiacylglycerol synthase from DAG and further is converted to DGDG through the digalactosyldiacylglycerol synthase (DGD) (Li and Yu 2018). The coordinated contents of MGDG and DGDG are therefore crucial not only for photosynthetic efficiency but also for the structural and functional stability of plastid membranes under both normal and stress conditions (Fujii et al. 2019, Li et al. 2020, Ge et al. 2022).

Other lipid classes are present within the cell, serving a metabolic or signaling function, such as DAG, which plays a central role in the biosynthesis of all lipids and membrane remodeling, ensuring that plant cells can adjust to changing physiological and environmental conditions (Mackender and Leech 1974). DAG represents a key intermediate of the acyl-CoA dependent Kennedy pathway, linking the synthesis of membrane lipids with metabolic (neutral) lipids (Cagliari et al. 2011). DAG in the endoplasmic reticulum can serve as a substrate for the synthesis of PC mediated by choline kinase (CK) and the reversal conversion of PC back to DAG catalyzed by non-specific phospholipase C (Peters et al. 2010, Guschina et al. 2014, Szymanski et al. 2014, Hölzl and Dörmann 2019, Lin et al. 2019, Makshakova et al. 2020). The other important non-structural lipid is triacylglycerol (TAG), which serves as a long-term energy reserve and which can be mobilized for lipid remodeling processes under environmental stresses such as low temperature or drought (Degenkolbe et al. 2012, Perlikowski et al. 2016, 2019, 2020, 2022, Yang and Benning 2018). The main source of TAG is the Kennedy pathway; the glycerol backbone required for TAG synthesis is derived from glycerol-3-phosphate (G3P). Subsequent acylation steps lead to the formation of phosphatidic acid, which is further dephosphorylated by phosphatidic acid phosphatases (PAH) and lipid phosphate phosphatase (LPP), yielding DAG. The terminal step of TAG biosynthesis involves the acetylation of DAG. This reaction is catalyzed either by acyl-CoA-dependent diacylglycerol acetyltransferase (DGAT) or by phospholipid:diacylglycerol acyltransferase (PDAT), which use membrane phospholipids as the acyl donor (Cagliari et al. 2011, Tan et al. 2018, Shomo et al. 2024). In parallel, TAG formation may also proceed via alternative acyltransferase activities mediated by phytyl ester synthase (PES) in chloroplasts, which utilizes fatty acids released during membrane lipid remodeling, particularly from galactoglycerolipids, thereby linking chloroplast membrane turnover with neutral lipid accumulation under stress conditions (Lippold et al. 2012).

Tall fescue (*Festuca arundinacea*) is a forage grass species with a high adaptive potential, enabling it to thrive across a wide range of climatic and soil conditions. Its ability to adjust metabolism to variable environmental factors is a critical determinant of its successful establishment in agricultural ecosystems. Moreover, it exhibits significant tolerance to abiotic stresses, including exposure to drought, salinity, and low temperatures, making it a valuable component of permanent grasslands, particularly in regions characterized by harsh climatic conditions (Brock et al. 1982, Kosmala et al. 2007, Kosmala et al. 2012, Pawłowicz et al. 2017, Pawłowicz et al. 2018, Augustyniak et al. 2018, Augustyniak et al. 2020, Perlikowski et al. 2019, Perlikowski et al. 2022, Perlikowski et al. 2023). Owing to these attributes, tall fescue could serve as a valuable model species for investigating frost tolerance and winter hardiness in forage grasses.

The objective of the current study is to investigate the physiological and biochemical mechanisms underlying plant frost tolerance, with a particular emphasis on the leaf lipidome of *F. arundinacea*. The frost tolerance was evaluated according to the LT_50_ parameter (temperature causing 50% of electrolyte leakage), which serves as a reliable physiological marker of plasma membrane functionality, providing quantitative insight into both the stability and structural integrity of cellular membranes under abiotic stress conditions. Furthermore, this parameter was proven several times to be strictly correlated with frost tolerance observed in *Festuca* species and their intergeneric hybrids with *Lolium* species (Kosmala et al. 2006, Kosmala et al. 2007, Kosmala et al. 2009, Bocian et al. 2011, Augustyniak et al. 2018). It is hypothesized that observed changes in *F. arundinacea* lipidome and the expression of genes coding proteins associated with lipid metabolism will correlate with the physiological indicators of plant performance and ability to acquire or lose frost tolerance during CA, DA and RA. To the best of our knowledge, this is the first comprehensive study focused on leaf lipidome remodeling under fluctuating temperatures, which are the constant attributes of winters in temperate climate, closely associated with climate change, and its influence on frost tolerance in forage grasses.

## Results

### Leaf physiology

To provide a physiological context for lipid remodeling during CA, DA, and RA, we analyzed parameters reflecting membrane stability, the level of oxidative stress, and photosynthetic performance in plant cells. All measurements were performed at five defined stages of the experiment (Fig. 1). The first stage involved analyses of control plants under 22°C; the second stage was pre-hardening the plants at 12°C for 7 days, with a 10-h photoperiod. Subsequently, the plants underwent 21 days of cold-acclimation, during which the temperature was maintained at 4°C during the day and 2°C at night, with the same photoperiod, and the plants were analyzed after 21 days of cold acclimation. After the CA, the DA process was initiated and lasted for 7 days, during which the temperature was raised to 12°C, while light conditions remained unchanged, and the plants were analyzed after one (DA-1) day and after seven (DA-7) days of de-acclimation. The final stage was RA, and it lasted for 21 days. During this phase, the plants were exposed to conditions of 4°C during the day and 2°C at night. During RA, plants were analyzed after three (RA-3) and 21 days (RA-21) of this process (Fig. 1). During the experiment, the light parameters were maintained constant, 10-h photoperiod and 200 *μ*mol m^2^s^−1^ PPFD (Fig. 1). Electrolyte leakage (LT_50_) was used as an indicator of frost tolerance and membrane integrity, while H₂O₂ content and MDA accumulation reflected the level of oxidative stress and lipid peroxidation. Chlorophyll fluorescence parameters were used to assess the efficiency of PSII and PSI in relation to thylakoid membrane function. For pre-acclimation (PA) and CA, the data are presented in relation to the control conditions. De-acclimation (DA-1 and DA-7) data are discussed in relation to CA, whereas re-acclimation (RA-3 and RA-21) data are discussed in relation to the DA-7 time point (Figs. 2 and 3). Representative plant phenotypes during the crucial steps of the experiment are shown in Supplementary Fig. S1.

**Figure 1:**
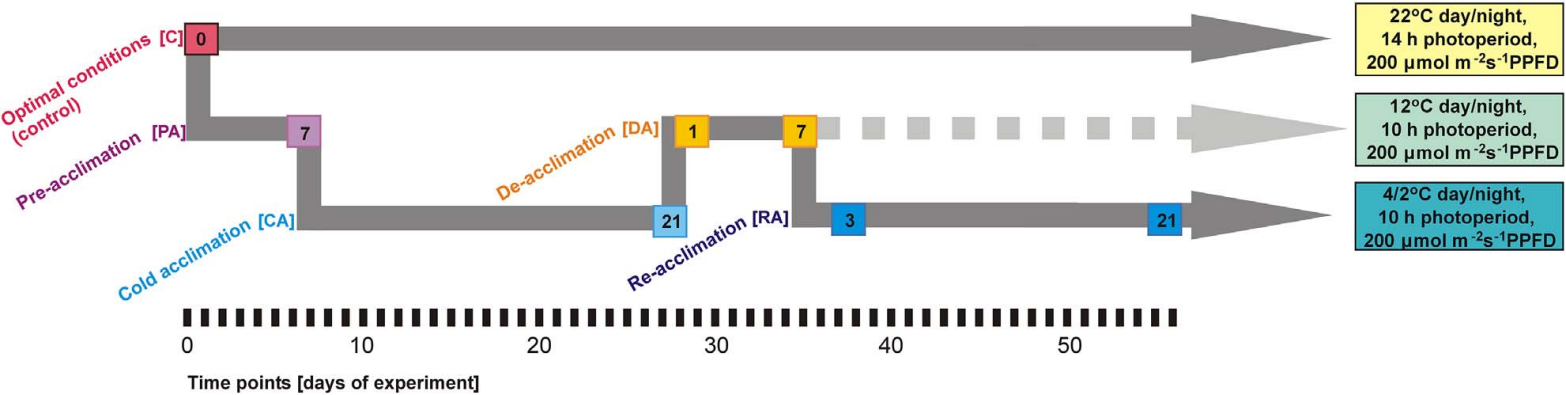
Timeline of the experiment performed on *Festuca arundinacea*, illustrating the control conditions (C) and four other phases of the experiment: PA, CA, DA, and RA. Control (C) represents the initial state of the experiment. CA represents the twenty-first day of cold acclimation. DA represents the 1st (DA-1) and 7th (DA-7) days of de-acclimation. RA represents the 3rd (RA-3) and 21st (RA-21) days of re-acclimation.

**Figure 2:**
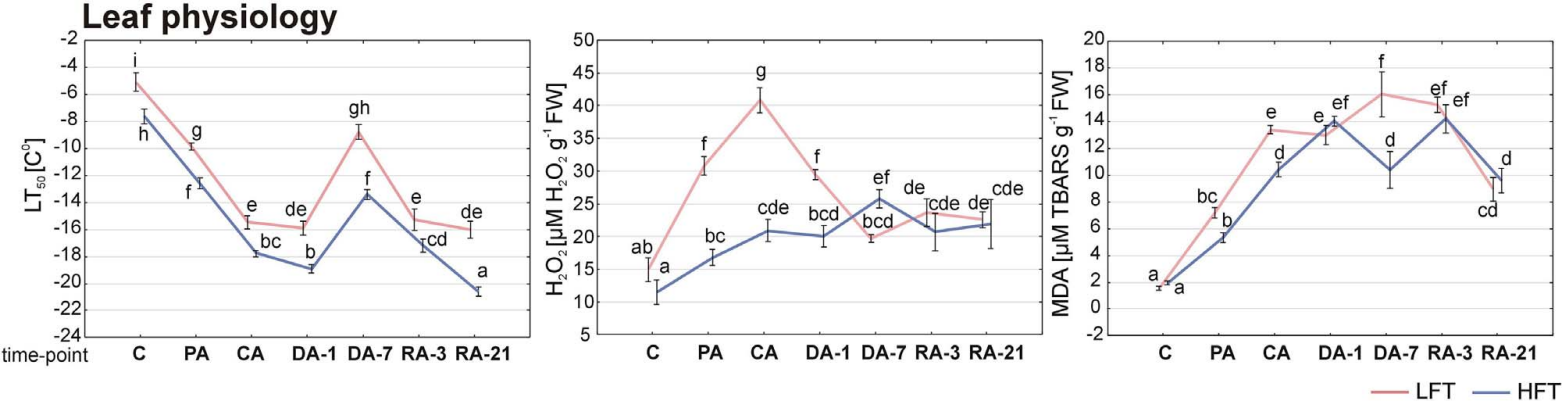
Physiological parameters analyzed in the LFT and the HFT genotypes of *F. arundinacea* measured at seven time points of the experiment: the control conditions (C), PA, CA, the 1st day of de-acclimation (DA-1), the 7th day of de-acclimation (DA-7), the 3rd day of re-acclimation (RA-3), and the 21st day of re-acclimation (RA-21). LT_50_ (temperature causing 50% electrolyte leakage), H_2_O_2_ content, and MDA content represented by thiobarbituric acid reactive substances (TBARS) content. Error bars represent standard errors of the means, and letters indicate groups that do not differ significantly at the significance level of *P* ≤ 0.05 (Fisher’s LSD test).

**Figure 3:**
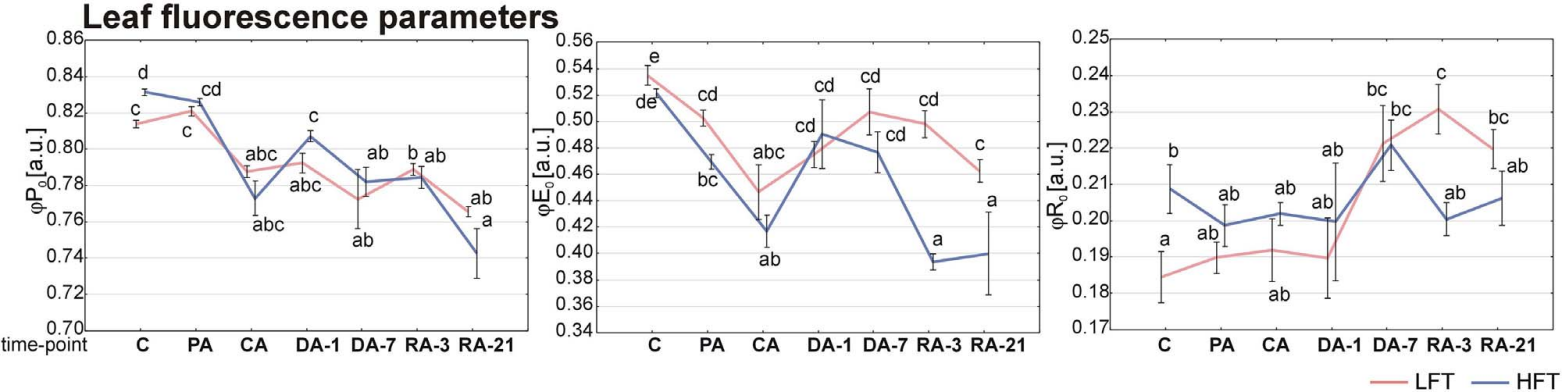
Chlorophyll fluorescence parameters analyzed in the LFT and the HFT genotypes of *F. arundinacea* measured at seven time points of the experiment: the control conditions (C), PA, CA, the 1st day of de-acclimation (DA-1), the 7th day of de-acclimation (DA-7), the 3rd day of re-acclimation (RA-3), and the 21st day of re-acclimation (RA-21). **φ*P***_**0**_ (the maximum quantum yield of primary PSII photochemistry at the onset of illumination (all PSII reaction centers open), **φ*E***_**0**_ (the quantum yield for electron transport beyond Q_A_^−^ at *t* ≈ 0) (all PSII reaction centers open), **φ*R***_**0**_ (the quantum yield for the reduction of PSI end acceptors per absorbed photon at *t* ≈ 0). Error bars represent standard errors of the means, and letters indicate groups that do not differ significantly at the significance level of *P* ≤ 0.05 (Fisher’s LSD test), a.u., auxiliary units.

### Cellular membrane stability

PA followed by CA induced a significant reduction in the temperature at which 50% electrolyte leakage (LT_50_) occurred in both analyzed genotypes (Fig. 2). However, LT_50_ values were consistently lower in the HFT genotype (−17.8°C), compared to the LFT genotype (−15.5°C). Observations at 1-DA revealed an inhibition of CA. The process of de-acclimation was clearly visible at DA-7, when the LT_50_ parameter increased in the LFT to the value −8.8°C and in the HFT to −13.4°C. However, the values of LT_50_ at DA-7 did not return to those observed under the control conditions. During RA, a decrease in LT_50_ values was revealed in both genotypes. In the HFT genotype, LT_50_ continued to decrease throughout the entire RA period, reaching values at RA-21 (−20.6°C) even lower than those recorded during CA. In the LFT genotype, at RA-3, LT_50_ decreased to the values observed during CA and then remained constant.

### Hydrogen peroxide (H₂O₂) content

A significant increase in H₂O₂ content during PA and its progression at CA was observed in both genotypes; however, the changes were more pronounced in the LFT genotype, compared to the control (Fig. 2). Furthermore, the level of H₂O₂ in the LFT genotype was higher than in the HFT genotype. DA revealed a reduction in H₂O₂ content in the LFT genotype relative to CA. In contrast, DA did not affect the HFT genotype with respect to H₂O₂ level, although statistically significant differences were noticed between DA-1 and DA-7. During RA, no significant changes in H₂O₂ content were observed in both analyzed genotypes, compared to DA, and no statistically significant differences between these genotypes were detected.

### Lipid peroxidation—MDA accumulation

A significant increase in MDA content was observed during PA and progressed at CA in both genotypes, compared to the control; however, the LFT genotype was characterized by a significantly higher level of MDA, compared to the HFT genotype during CA (Fig. 2). After 1 day of de-acclimation in the HFT genotype, an increase in MDA level was noticed, but after 7 days of de-acclimation, it returned to the value comparable to that observed during CA. In contrast, no change of MDA content was shown in the LFT genotype at DA-1, while a significant increase was revealed at DA-7. Moreover, the level of MDA in the LFT genotype was significantly higher compared to the HFT genotype at this time point. During RA, no differences in MDA content were observed in the LFT genotype at the early stage of RA, compared to DA, but a significant decrease in this parameter was observed after 21 days of re-acclimation, reaching the values observed in PA. In contrast, the HFT genotype demonstrated an increase in MDA level at the early stage of RA, reaching a value comparable to that recorded at DA-1, but after the full RA process it returned to the value observed at DA-7 or CA.

### Chlorophyll fluorescence

During the experiment, considerable differences in chlorophyll fluorescence parameters were observed between treatments and genotypes (Fig. 3). Maximum quantum yield of primary PSII photochemistry (φ*P*_0_ also known as *F_v_/F_m_*) decreased under CA in the HFT genotype, relative to the control, indicating a loss of PSII photochemical efficiency in cold in this plant (Fig. 3). However, during DA and RA, φ*P*_0_ in the HFT did not change significantly compared to CA. On the other hand, the value of φ*P*_0_ in the case of the LFT genotype was more stable during the whole experiment; however, at DA-7, RA-3, and RA-21, a significantly lower value of φ*P*_0_ compared to the control was observed. Differences between the LFT and the HFT genotypes were only visible in the control time point. Quantum yield for electron transport beyond QA^−^ (φ*E*_0_) declined through PA and progressed in CA in both genotypes, and then it increased during DA, but significantly only in the HFT genotype (Fig. 3). In RA, QA^−^ dropped in the HFT to the value observed during CA, but it remained stable, compared to DA, in the LFT genotype. The clear differences between the analyzed plants were observed during the RA process. The pattern of changes in φ*R*_0_ (quantum yield for the reduction of PSI end acceptors) differed from the PSII-dependent parameters (Fig. 3). This parameter did not change significantly during the whole experiment in the HFT genotype, but its higher values in the case of the LFT genotype were observed during RA, compared to the control time point. The clear differences between the two analyzed genotypes were visible in the control conditions and after 3 days of re-acclimation.

### Accumulation profiles of glycerolipid classes

In order to investigate the specific lipid remodeling strategies that contribute to frost tolerance, we analyzed the accumulation profiles of phosphoglycerolipids: PE and PC, galactoglycerolipids: MGDG and DGDG, and metabolic lipids: DAG and TAG. We also analyzed their lipid species composition and unsaturation level according to the double bond index (DBI). All measurements were performed at defined time points: control—Day 0 at 22°C, after 7 days of PA in 12°C, after 21 days of cold acclimation in 4°C, on the 1st and 7th day of de-acclimation in 12°C and on the 3rd and 21st day of re-acclimation in 4°C (Fig. 1). For PA and CA, data are discussed in comparison to the control conditions. De-acclimation (DA-1 and DA-7) values are discussed in relation to CA, whereas re-acclimation (RA-3 and RA-21) values are discussed in comparison to DA-7 (Figs. 4–6, Supplementary Table S1).

**Figure 4:**
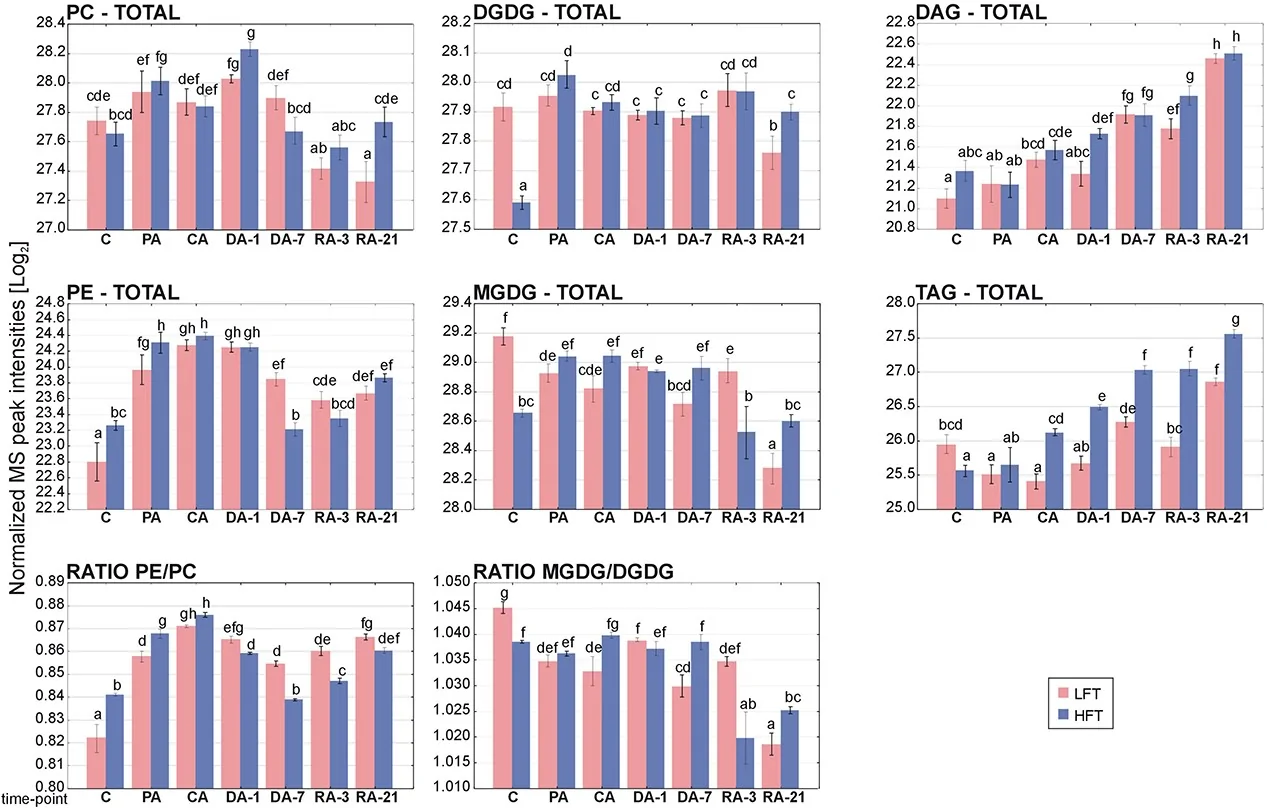
Accumulation levels of analyzed lipid classes in the LFT and the HFT genotypes of *F. arundinacea* measured at seven time points of the experiment: the control conditions (C), PA, CA, the 1st day of de-acclimation (DA-1), the 7th day of de-acclimation (DA-7), the 3rd day of re-acclimation (RA-3), and the 21st day of re-acclimation (RA-21). Error bars represent standard errors of the means. Bars represent mean values of the log₂-transformed sum of all lipid species mass spectral peak intensities within a particular lipid class. Letters indicate groups of means that do not differ significantly at the significance level of *P* ≤ 0.05 (Fisher’s LSD test).

**Figure 5:**
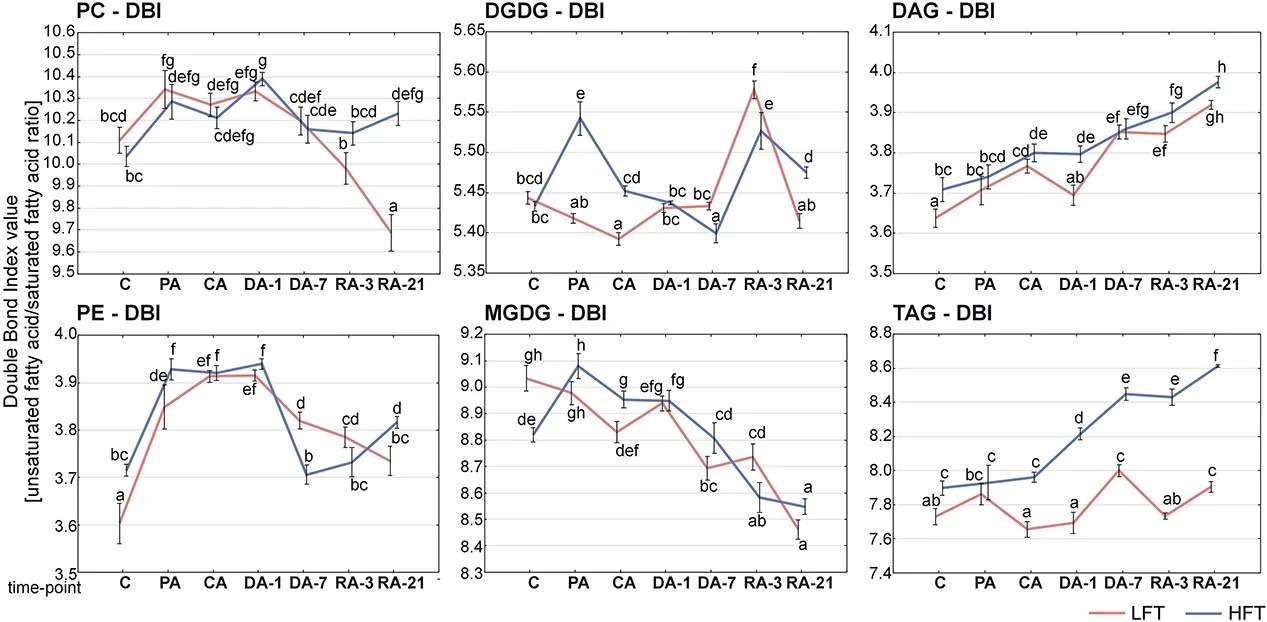
The DBI of analyzed lipid classes in the LFT and the HFT genotypes of *F. arundinacea* calculated at seven time points of the experiment: the control conditions (C), PA, CA, the 1st day of de-acclimation (DA-1), the 7th day of de-acclimation (DA-7), the 3rd day of re-acclimation (RA-3), and the 21st day of re-acclimation (RA-21). Letters indicate groups of means that do not differ significantly at the significance level of *P* ≤ 0.05 (Fisher’s LSD test).

**Figure 6:**
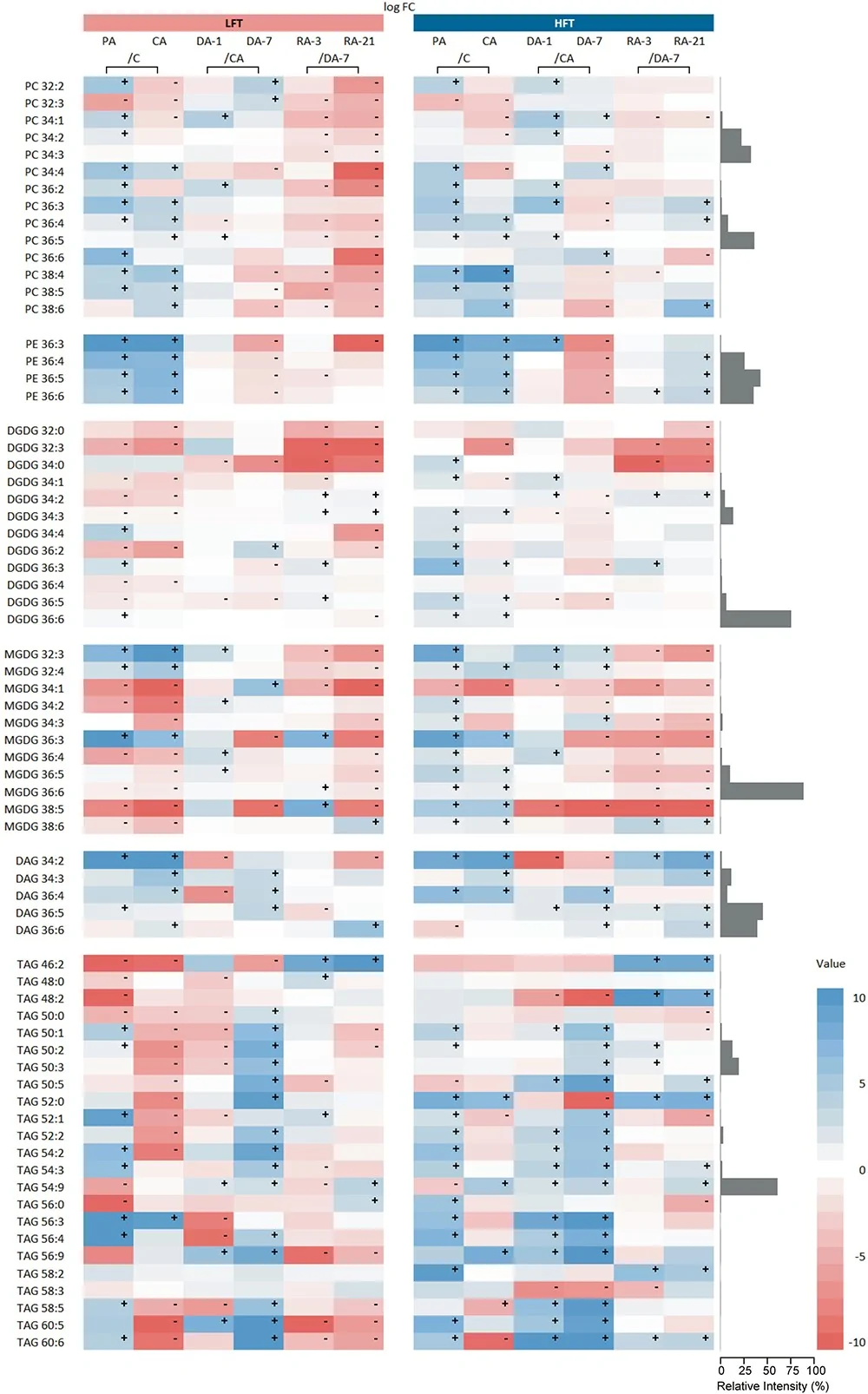
The accumulation of lipid species of analyzed lipid classes in the LFT and the HFT genotypes of *F. arundinacea* measured at six time points of the experiment: PA, CA, the 1st day of de-acclimation (DA-1), the 7th day of de-acclimation (DA-7), the 3rd day of re-acclimation (RA-3), and the 21st day of re-acclimation (RA-21). The lipid accumulation at PA and CA are shown in relation to the control conditions (C), at DA-1 and DA-7 in relation to CA, and at RA-3 and RA-21 in relation to DA-7. Plus (+) and minus (−) indicate significant differences in accumulation at *P* ≤ 0.05 (Fisher’s LSD test), compared to the reference time point. Grey bars represent the percentage contribution of particular lipid species within its group.

### Phosphoglycerolipids composition

A significant increase in total PC content was observed during PA only in the HFT genotype, compared to the control conditions. In both genotypes, in PA, we could observe an increase in total PC unsaturation level, which returned to the control values during CA (Fig. 5). The greatest contribution to the total PC accumulation level had the lipids with the highest abundance: PC 34:2, 34:3, and 36:5 (Fig. 6). The particular PC lipid species accumulation profiles revealed that most of the PC lipids, especially those rich in longer and polyunsaturated acyl chains, were significantly accumulated during the whole CA period in both genotypes, including the most abundant PC 36:5 lipid (Fig. 6). In both genotypes, PA induced accumulation of all highly unsaturated PE lipids, which were further elevated during CA; however, it is worth noting that under the control and PA conditions, a higher level of unsaturated PE was observed in the HFT genotype (Figs. 4 and 5). Among the identified PE lipids, the most abundant were those containing 36:4, 36:5, and 36:6 acyl chains (Fig. 6). The increase of PE accumulation also had a significant impact on the increased PE/PC ratio in both genotypes (Fig. 4).

DA had a significant impact exclusively on the HFT genotype, where accumulation of total PC increased at DA-1 and subsequently returned to the level observed during CA at DA-7 (Fig. 4). The increase in the accumulation of several lipid species with short and lower unsaturation levels was responsible for this situation (Fig. 6). However, this did not generally affect the DBI of PC, compared to CA (Fig. 5). However, we could observe that both genotypes significantly accumulated PC 36:5, but it was observed only at the DA-1 time point. During the DA, a decline in the total PE level was also observed in both genotypes; however, it only reached the control value in the HFT genotype. This phenomenon was visible after 7 days of de-acclimation. Moreover, the decrease in all PE species was observed at DA-7 in both genotypes, compared to CA (Fig. 6). This negatively impacted PE unsaturation level and PE/PC ratio (Figs. 4 and 5).

During RA, we could observe a significant decrease in total PC accumulation, but only in the LFT genotype, when compared to DA conditions (Fig. 4). This was also followed by a decrease in DBI in this genotype (Fig. 5). Accumulation levels of almost all PC species displayed a significant decrease at RA-21, compared to DA-7 in the LFT genotype (Fig. 6). RA also had a significant impact on PE accumulation, but only in the HFT genotype, where an increase was revealed on its 21st day (Fig. 4). This increase coincided with a significant accumulation of PE species 36:4, 36:5, and 36:6 (compared to DA-7) (Fig. 6), which together constituted the major proportion of the total PE pool and also positively affected DBI of PE at RA-21 (Fig. 5). However, no significant differences at that time point, compared to DA, were observed in the LFT genotype (Fig. 4). That said, a decreasing level of DBI in the LFT genotype could be observed (Fig. 5). What is interesting is that the differences in PC and PE accumulation profiles between the genotypes resulted in a similar increase in PE/PC ratio during RA (Fig. 4).

### Galactoglycerolipids composition

CA had a significant positive impact on total DGDG accumulation only in the HFT genotype (Fig. 4). That was mainly due to a higher accumulation level of the most abundant DGDG 36:6. In the LFT genotype, this DGDG species was only visibly accumulated at PA (Fig. 6). On the other hand, the DBI value for DGDG in the HFT genotype also only significantly increased during PA, while in the LFT genotype it decreased after full CA, compared to the control (Fig. 5). The MGDG accumulation in both genotypes was significantly affected by CA (Fig. 4). The HFT genotype showed an increased accumulation of total MGDG, whereas the LFT demonstrated a decrease in its accumulation compared to the control (Fig. 4). This trait was most highly affected by the most abundant MGDG 36:6 (Fig. 6). For most MGDG species, a significant decrease was observed in the LFT genotype during CA, compared to the control. By contrast, most of the highly unsaturated MGDGs exhibited increased accumulation in the HFT genotype during PA and CA (Fig. 6). These profiles were reflected in the MGDG unsaturation level, which increased in the HFT genotype and decreased in the LFT genotype under CA (Fig. 5). The MGDG/DGDG ratio also significantly decreased during CA, but only in the LFT genotype (Fig. 4).

No significant changes in total DGDG accumulation were demonstrated during the DA, compared to CA in either genotype (Fig. 4). However, the DA process itself had a significant effect on DGDG DBI in both genotypes, resulting in an increase of DBI in the LFT genotype and a decrease in the HFT genotype (Fig. 5). During DA, no significant changes in total MGDG accumulation were observed, relative to CA in either genotype (Fig. 4), despite changes in the accumulation of low abundant individual MGDG species (Fig. 6). A significant reduction in MGDG unsaturation level was, however, revealed in both genotypes (Fig. 5). Relevant changes in the MGDG/DGDG ratio were observed exclusively in the LFT genotype; however, only at DA-1 was a significantly higher ratio observed, followed by a decline to levels comparable to those recorded during CA (Fig. 4).

During RA, no differences were observed in total DGDG accumulation in the HFT genotype compared to the DA-7 time point (Fig. 4). RA had a significant impact exclusively on the LFT genotype, where it resulted in a decrease in total DGDG levels at RA-21 (Fig. 4). In both genotypes, an increase in DGDG unsaturation levels was observed at RA-3, followed by a decrease at RA-21, but the HFT genotype was characterized by higher DGDG unsaturation level at this timepoint (Fig. 5). RA resulted in a significant decline in total MGDG in both genotypes; however, this decrease occurred as early as on the 3rd day of re-acclimation in the HFT genotype, while in the LFT genotype it was observed only on the 21st day of re-acclimation for most of the MGDG lipid species (Figs. 4 and 6). A reduction of MGDG unsaturation level was observed during RA in both genotypes (Fig. 5). During RA, a decrease in the MGDG/DGDG ratio was observed in both genotypes; however, in the LFT genotype, it was visible after 21 days of re-acclimation, while in the HFT already after 3 days of re-acclimation (Fig. 4).

### A metabolic lipid composition

During CA, a significant increase in DAG accumulation, compared to the control conditions, was only observed in the LFT genotype, but these two genotypes did not differ significantly with respect to DAG content at this time point (Fig. 4). However, an increase in the level of DAG unsaturation was observed in both genotypes in response to CA (Fig. 5). Accumulation of TAG responded to the CA process in both studied genotypes (Fig. 4). An increase was observed in the HFT genotype, whereas a decrease occurred in the LFT genotype also during PA. The highest impact on TAG accumulation was displayed by TAG 54:9, but prominent changes were also observed for TAG 50:2 and TAG 50:3 (Fig. 6). During CA, the LFT genotype displayed a reduced abundance of most identified TAG lipid species compared to the control (Fig. 6). However, the CA process did not significantly affect the degree of TAG unsaturation in either genotype (Fig. 5).

Compared to CA, DA affected both genotypes, inducing higher accumulation of DAG after 7 days of de-acclimation (Fig. 4). In the LFT genotype, DBI decreased on the 1st day of de-acclimation, relative to CA, and subsequently increased on the 7th day of de-acclimation, exceeding the levels observed during CA. In contrast, the HFT genotype showed no significant changes during this phase. During DA, both genotypes exhibited an increase in TAG accumulation; however, the increase was earlier and significantly higher in the HFT genotype. In the case of the LFT genotype, these changes became evident only at DA-7. At DA-7 an increase in the abundance of most TAG species, especially unsaturated TAGs, was observed in both genotypes (Figs. 5 and 6, Supplementary Table S1).

RA revealed further accumulation of highly unsaturated DAG in the LFT and HFT genotypes, but only after 21 days of re-acclimation (Figs. 4 and 5). In the LFT genotype, only DAG 36:6 increased in accumulation at this time point (Fig. 6). Additionally, further accumulation of unsaturated TAG was observed in the HFT genotype but only at RA-21, while the LFT genotype first exhibited a decrease in unsaturated TAG content during the early stage of RA, but this was reverted, and a significant increase in total TAG accumulation was observed in this genotype at RA-21 (mainly due to TAG 46:2, 54:9, 56:0) (Figs. 4–6). It could additionally be observed that the HFT genotype was characterized by significantly higher TAG DBI, compared to the LFT genotype, starting from CA (Fig. 5).

### Transcriptome analysis

RNA sequencing generated a total of 2 927 860 132 high-quality clean reads, with base quality scores exceeding Q20 > 97% and Q30 > 94%. After quality control procedures, including adapter removal and trimming of low-quality bases, the remaining reads demonstrated even higher quality metrics (Q20 > 99%, Q30 > 97%). Subsequent analysis identified 33 126 and 28 791 differentially expressed genes (DEGs) (*P* < 0.05, |log₂FC| ≥ 2) in the LFT and HFT genotypes, respectively, based on the comparisons of each experimental time point to the control values. In the LFT genotype, the number of upregulated DEGs (18 501) exceeded that of downregulated (14 625). In contrast, in the HFT genotype, a greater number of downregulated genes (15 391) was observed, compared to upregulated ones (13 400) (Supplementary Fig. S2). These DEGs were classified into 13 functional categories and associated metabolic pathways based on the enzymatic functions of proteins encoded by the corresponding genes and performed based on GO and the KEGG database (Supplementary Fig. S3). A total of 24 389 reads were obtained from the sequencing and subsequently subjected to quality filtering and differential expression analysis. Applying a selection threshold of |log₂FC| ≥ 2 resulted in the identification of 14 811 DEGs. These DEGs were then annotated against the KEGG database, allowing identification of 4121 genes associated with defined metabolic pathways. From this dataset, genes involved in lipid metabolism were extracted for further analysis. Among them, 226 DEGs related to lipid biosynthesis and degradation met the |log₂FC| ≥ 2 criteria. An additional filtering step using a threshold of |log₂FC| ≥ 3 identified 91 DEGs directly implicated in the biosynthesis and degradation of lipids essential for the PC, PE, MGDG, DGDG, DAG, and TAG pathways (Fig. 7, Supplementary Table S2). These DEGs exhibited numerous transcriptional changes during PA and CA, compared to the control, during de-acclimation after 1 and 7 days, relative to CA, and after 3 and 21 days of re-acclimation, relative to 7th day of de-acclimation (Figs. 7 and 8) are discussed in the Discussion section. The expression level of each transcript was used here to deduce potential roles of the enzymes responsible for lipid metabolism.

**Figure 7:**
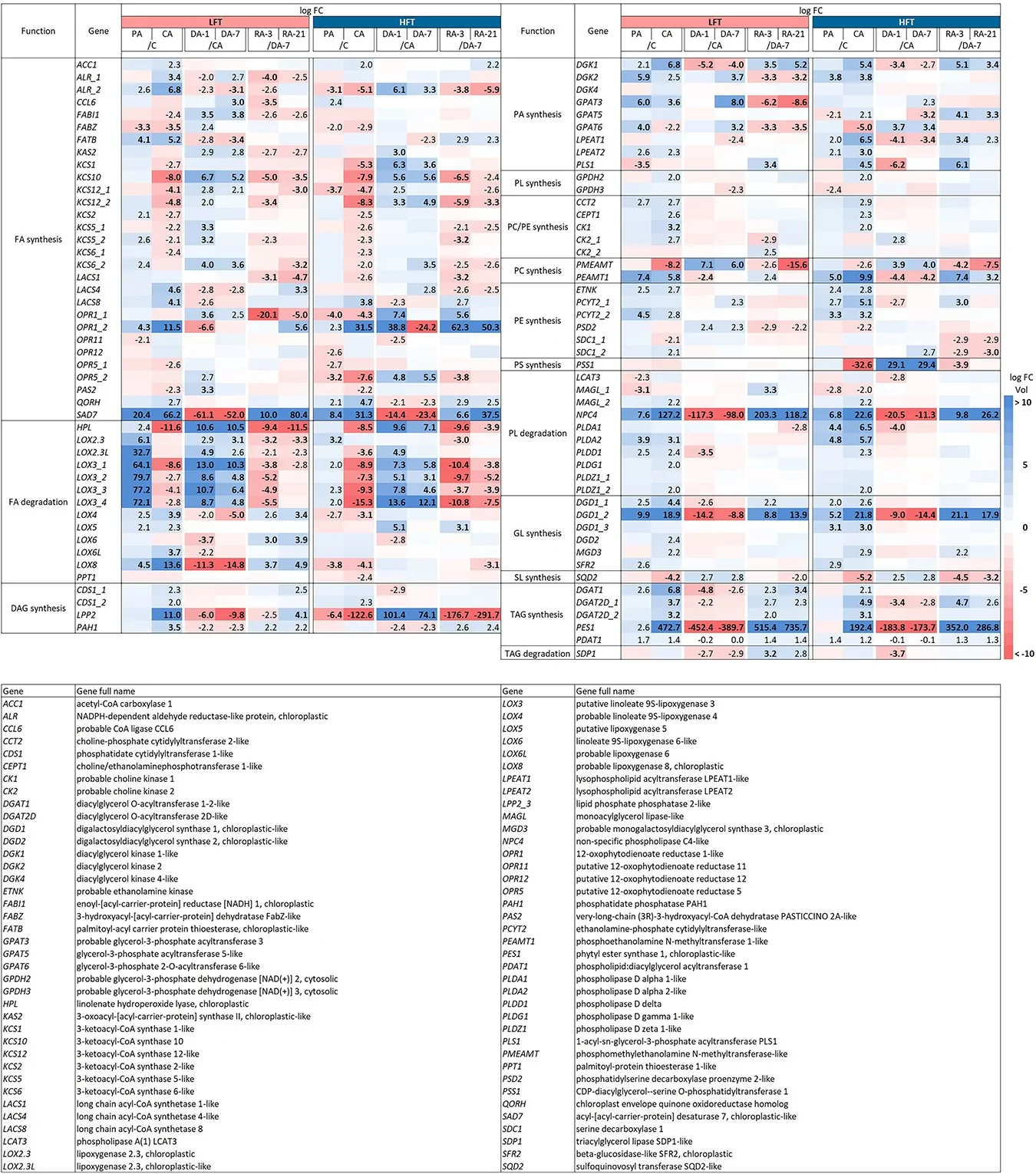
Fold changes of transcripts associated with lipid metabolism of the LFT and the HFT genotypes of *F. arundinacea* measured at six time points of the experiment: PA, CA, the 1st day of de-acclimation (DA-1), the 7th day of de-acclimation (DA-7), the 3rd day of re-acclimation (RA-3), and the 21st day of re-acclimation (RA-21). The PA and CA are shown in relation to the control conditions (C), DA-1 and DA-7 in relation to CA, and RA-3 and RA-21 in relation to DA-7. DEGs involved in lipid biosynthesis and metabolism were identified using a discrimination threshold of |log₂FC| ≥ 2.

**Figure 8:**
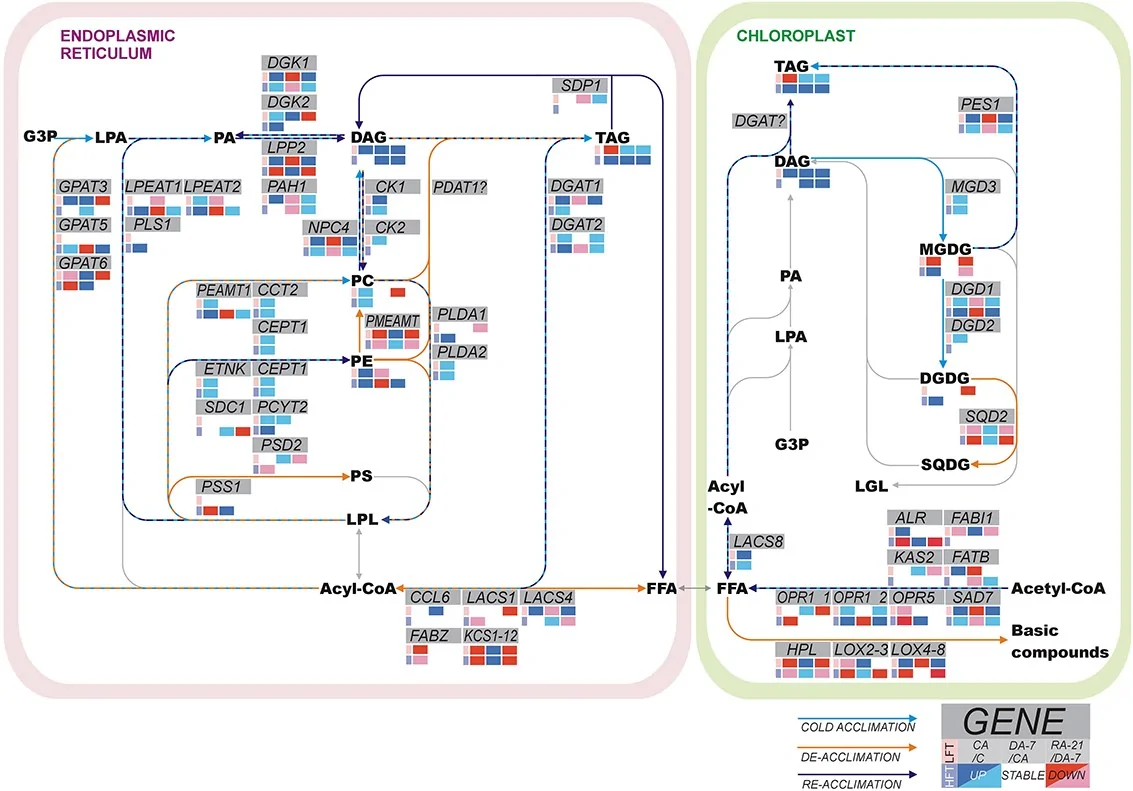
A representative model of lipidome rearrangements induced by CA, DA, and RA processes in the LFT (pink boxes) and the HFT (violet boxes) genotypes of *F. arundinacea*, based on identified lipid and DEG accumulation profiles. Blue boxes indicate upregulated genes (deep blue—more significant changes, light blue—less significant changes), red boxes indicate downregulated genes (deep red—more significant changes, pink—less significant changes), and white boxes represent no significant change. The first box represents the CA process, compared to the control (C); the second box represents 7th day of the de-acclimation process (DA-7), compared to CA, and the third box represents the 21st day of the re-acclimation process (RA-21), compared to DA-7. Light blue arrows indicate processes that are promoted under CA, orange arrows indicate processes that are promoted under DA, and deep blue arrows indicate processes that are promoted under re-acclimation. Nomenclature not present in the manuscript text: G3P, glycerol-3-phosphate; LPA, lysophosphatidic acid; PA, phosphatidic acid; LPL, lysophosphoglycerolipid; FFA, free fatty acid; PS, phosphatidylserine; SQDG, sulfoquinovosyl diacylglycerol; LGL, lysogalactoglycerolipid.

The similarity between samples was assessed using Pearson’s correlation coefficient calculated from log-transformed expression values. The correlation coefficient (*r*) ranges from −1 to 1, where values closer to 1 indicate higher similarity between samples. The analysis revealed consistently high correlation among biological replicates, confirming the reliability of the RNA-seq data. In both the HFT and LFT genotypes, all replicates showed correlation values above 0.96. However, differences across time points were also observed. Samples collected within the same experimental stage clustered together, while those from distinct stages displayed opposite results, with lower correlation values (Supplementary Fig. S4).

## Discussion

Our results clearly showed that both analyzed genotypes of *F. arundinacea* were able to acquire frost tolerance during CA. However, the HFT genotype was characterized to better tolerate freezing temperatures across all experimental phases (Fig. 2). These observations are consistent with previous studies on forage grasses such as *Lolium perenne, F. pratensis*, introgression forms of *Lolium multiflorum × F. arundinacea*, and the model species *Arabidopsis thaliana*, which also reported increases in frost tolerance during CA. Moreover, the differences between more and less tolerant genotypes remained evident throughout the experiments (Kosmala et al. 2009, Bocian et al. 2011, Zuther et al. 2015, Augustyniak et al. 2018, Augustyniak et al. 2020).

During de-acclimation, the observed genotype differences in the levels of frost tolerance still remained. However, this phase revealed other important features of the de-acclimation process in *F. arundinacea*. It was observed that 1-day exposure to higher temperatures was insufficient to cause the loss of frost tolerance in the analyzed plants. Indeed, a significant loss of frost tolerance was observed only after 7 days of de-acclimation (Fig. 2). However, even such a relatively long exposure to higher temperature was not sufficient to fully de-acclimate analyzed plants. Similar patterns have been reported in winter wheat (*Triticum aestivum*); 7 days of de-acclimation resulted in a significant reduction of frost tolerance, although a complete reversal of hardiness was not observed (Vaitkevičiūtė et al. 2022). Similarly, in winter barley (*Hordeum vulgare*), following three weeks of CA, 7 days of de-acclimation increased LT_50_ values in all tested lines, confirming the occurrence of DA, yet full loss of frost tolerance was not achieved (Wójcik-Jagła and Rapacz 2023). In contrast, in different plant species, alterations in frost tolerance can occur even within a shorter time frame. In Commerson’s wild potato (*Solanum commersonii*), 1 day of exposure to warm conditions was sufficient to reverse most of the physiological adjustments induced by CA, with initial changes observed within 2–3 h of exposure to higher temperature (Chen and Li 1980). Similar rapid responses have been reported in *A. thaliana*, where LT_50_ values increased significantly after just 1 day of de-acclimation, reflecting a partial loss of frost tolerance. In the same study, a cold-tolerant accession exhibited an 81% reduction of additional frost tolerance acquired during CA following 3 days of de-acclimation (Zuther et al. 2015). These findings suggest that species exhibiting higher susceptibility to abiotic stresses initiate DA more rapidly than perennial grasses such as *F. arundinacea.* Similar dynamics have been observed in turf grass species. In annual bluegrass (*Poa annua*), decreases in frost tolerance were detected in all ecotypes after a single day of de-acclimation conditions. Extended exposure to higher temperatures for 5 days resulted in near-complete DA in both frost-tolerant and frost-sensitive genotypes. Creeping bentgrass (*Agrostis stolonifera*), a species with higher tolerance to abiotic stresses compared to *P. annua*, did not achieve full DA under the same conditions (Hoffman et al. 2014). Despite increasing attention being paid to DA process in plants, most studies have been restricted to *A. thaliana* (Zuther et al. 2015, Juszczak et al. 2016) and other dicots such as oilseed rape (*Brassica napus*) (Rapacz 2002a, 2002b, Stachurska et al. 2022, Rys et al. 2025)*,* but even in these studies, there was a striking variability in both the timing of the DA process and the temperature applied in the experiments. By contrast, the knowledge on the physiological nature of the DA process in monocots is limited and restricted mostly to cereals (Rapacz et al. 2022, Wójcik-Jagła and Rapacz 2023, Pociecha et al. 2025).

During RA, our data revealed that 3-day exposure to acclimating temperature was sufficient for both plants to restore the level of frost tolerance observed after a full 21 days of the first CA (Fig. 2). This phenomenon may reflect preserved metabolomic modifications and physiological stress memory, which allows plants to re-acclimate faster and reach higher frost tolerance during subsequent cold stress. This memory can persist for several days, affecting both the timing and intensity of the response, with rapid RA occurring when the primed state is maintained, while prolonged DA can lead to its loss and slower or incomplete recovery (Zuther et al. 2019, Leuendorf et al. 2020). This strategy, in which the HFT genotype progressively achieved even higher frost tolerance after 21 days of re-acclimation, was not observed in the LFT genotype. These observations reflect genotype-specific differences in the dynamics of RA, with the HFT genotype potentially adapting a more efficient response to fluctuating temperatures, including restoration of membrane stability following a temporal episode of higher temperature in DA conditions. Similar genotype-dependent RA strategies have been reported in *T. aestivum*, where 7 days of de-acclimation followed by 14 days of re-acclimation enabled most genotypes to restore frost tolerance (Vaitkevičiūtė et al. 2022). In *H. vulgare*, long-term CA (up to 112 days) followed by de-acclimation during 5 or 14 days and subsequent RA restored frost tolerance and LT_50_ to levels comparable to those achieved during CA (Vítámvás and Prásil 2008). In *A. thaliana*, research on CA has shown that plants previously exposed to low temperatures were able to restore their frost tolerance more quickly when re-exposed to low temperatures, reflecting the presence of cold memory, which allowed for effective and rapid RA (Zuther et al. 2019, Leuendorf et al. 2020). Taken together, these findings suggest that the analyzed plants had the capacity to restore frost tolerance, with the extent of recovery depending on the genotype. The observed consistently higher frost tolerance noticed in the HFT genotype could suggest that this genotype maintained higher membrane structural integrity, providing a significant advantage under low-temperature stress. These observations give a foundation for the subsequent analysis of lipid remodeling.

### The accumulation of unsaturated PE lipids is associated with the acquisition or loss of frost tolerance in *F. arundinacea*

In our study, CA resulted in the rearrangements in PE levels mainly associated with an increase of highly unsaturated PE content in both genotypes (Figs. 4 and 8). Simultaneously, PC content did not change in a similar manner, leading to an increase in the deduced PE/PC ratio. Even though overall PC levels remained stable during CA in both genotypes, accumulation of highly unsaturated PC lipids with longer chains was still visible in both genotypes (Figs. 4 and 6). Similar observations of an increase in unsaturated PC and PE species were also observed in *A. thaliana* under low temperature stress, being coupled with an increase in the deduced PE/PC ratio (Welti et al. 2002). In addition, in *A. thaliana* CA was associated with an increase in the proportion of diunsaturated PC and PE species, which may also suggest a shift towards a higher level of unsaturation of membrane lipids (Uemura et al. 1995). By contrast, in most previous studies, CA was associated with a simultaneous increase in both PE and PC contents (Welti et al. 2002, Fontanier et al. 2020). PE is a lipid, which favors the formation of non-lamellar structures such as the hexagonal II (HII) phase. Its small ethanolamine head group, which provides a cone-shaped structure, drives inward membrane curvature and promotes membrane fluidity. In contrast, PC favors the formation of stable lamellar structures in membranes. Its larger choline head group and cylindrical shape enable efficient packing within the bilayer, contributing to increased membrane rigidity (McIntosh 1996). Although the HII phase formed by PE enhances membrane fluidity, it does not provide the same level of functional organization as the lamellar bilayer. Therefore, the balance between lamellar and non-lamellar lipids is crucial for maintaining both fluidity and functional integrity of membranes (Cullis and de Kruijff 1979, Uemura et al. 1995). Under low-temperature stress, the lipid bilayer undergoes reduced fluidity and increased rigidity, which can lead to phase transitions, loss of membrane integrity, and cellular leakage. Consequently, adjustments in lipid composition, as well as in the activity of enzymes involved in lipid biosynthesis and turnover, are crucial for plant CA. Such remodeling enables the maintenance of membrane fluidity within a functional range, thereby preserving membrane stability and associated cellular processes under stress conditions (Uemura and Steponkus 1999, Mazur et al. 2020). In our work, the remodeling of PE/PC during CA was positively correlated with up-regulation of several genes associated with PE and PC synthesis, for example, the genes coding for choline-phosphate cytidylyltransferase (*CCT2*), choline kinases (*CK1, CK2*), choline/ethanolamine phosphotransferase (*CEPT1*), phosphoethanolamine *N*-methyltransferase 1 (*PEAMT1*), ethanolamine kinase (*ETNK*), and ethanolamine-phosphate cytidylyltransferase (*PCYT2*) (Figs. 7 and 8)*.* The observed increase in PE may reflect the coordinated up-regulation of *ETNK, PCYT2*, and *CEPT1* in both genotypes, representing ethanolamine biosynthesis pathway leading to PE synthesis (Dorighello et al. 2023). The observed expression of these genes were further maintained through DA and RA in both genotypes. In the LFT genotype only *PCYT2_2* gene was up-regulated but at slightly lower level, compared to the HFT, whereas in the HFT genotype both, *PCYT2_1* and *PCYT2_2*, were expressed (Figs. 7 and 8). In parallel, *PEAMT1* might provide phosphocholine, supplying substrate for PC synthesis, while *CEPT1* could additionally contribute depending on substrate availability (Dorighello et al. 2023). Previous reports have indicated that *PEAMT* homologs were transcriptionally upregulated during CA in *T. aestivum*, promoting increased PC biosynthesis under low-temperature stress conditions (Charron et al. 2002).

An important intermediate in membrane lipid remodeling and a direct precursor for lipid synthesis is DAG, being formed either *de novo* from phosphoric acid or through hydrolysis of membrane lipids such as PC (Cagliari et al. 2011, Szymanski et al. 2014, Bates and Shockey 2025). In the present study, both genotypes showed a very similar pattern of gradual DAG accumulation during the experiment. DAG remodeling during CA, DA, and RA was likely associated with changes in transcript levels of several genes, including the genes coding: glycerol-3-phosphate acyltransferases (*GPAT3, GPAT5,* and *GPAT6*), lysophospholipid acyltransferases (*LPEAT1, LPEAT2*), 1-acyl-sn-glycerol-3-phosphate acyltransferase (*PLS1*), phosphatidate phosphatase (*PAH1*), *DGK1, DGK2*, lipid phosphate phosphates (*LPP2*) and non-specific phospholipase C4 (*NPC4*). During CA, a significant increase of DAG, compared to the control values, was observed only in the LFT genotype (Figs. 4 and 8). This genotype was characterized by a significantly higher expression of *LPP2, PAH1*, and *GPAT3* under CA (Figs. 7 and 8), which might coincide with increased DAG accumulation and might reflect changes in early steps of *de novo* lipid synthesis (Cagliari et al. 2011). Moreover, the lack of significant increase in PC and higher DAG level could reflect the concurrent up-regulation of *NPC4* coding the enzyme, which catalyzes PC turnover to DAG during PA and further enhances it during CA (Figs. 7 and 8). Although *NPC4* gene has not been previously studied under cold stress, its role in DAG regulation under other stress conditions indicates a potential involvement in stress-related lipid remodeling (Peters et al. 2010, Fan et al. 2023). In the HFT genotype, *LPEAT1, LPEAT2*, and *PLS1* were induced, what might be related to lysophospholipid remodeling potentially linked to DAG metabolism (Jasieniecka-Gazarkiewicz et al. 2021), but no significant increase of total DAG, compared to the control, was observed. At the same time*, DGK1* was also highly expressed in both genotypes; however, genotypic differences related to the gene variants could be observed (Figs. 7 and 8). In plants, DAG is rapidly phosphorylated into PA by DGK, and our results suggest that in the LFT genotype this activity might have been balanced by increased *PAH1* and *LPP2* expression, which might facilitate DAG accumulation (Arisz et al. 2018). DGK activity has been proposed as an important indicator of freezing stress response in *A. thaliana* (Tan et al. 2018). Together with DAG remodeling, transcriptional changes in long-chain acyl-CoA synthetases (*LACSs*) might indicate coordinated regulation of fatty acid metabolism. Additionally, LACS4, localized in the endoplasmic reticulum, which gene was specifically upregulated in the LFT genotype, might be linked to the conversion of free fatty acids into acyl-CoA for subsequent DAG synthesis. LACS are known to catalyze the formation of acyl-CoA from free fatty acids, providing substrates for lipids synthesis, and preventing cytotoxic accumulation of free fatty acids (Cagliari et al. 2011, Jessen et al. 2015). PC biosynthesis might also be supported by *CK1* in both genotypes and by *CK2* in the LFT, coding the enzymes catalyzing the conversion of DAG to PC via the CDP-choline pathway. Such coordinated activity of many genes might help to maintain relatively stable PC levels during CA, balancing its synthesis and degradation (Figs. 7 and 8) (Sagar and Singh 2021). Despite genotype-specific differences at the transcription level of *PEAMT1*, no significant differences in total PC and PE were observed in both genotypes (Figs. 7 and 8). However, it is possible that higher expression of *PCYT2* in the HFT genotype under CA might be associated with higher level of PE observed in this genotype during PA (Figs. 7 and 8).

In the present study, it was observed that DA also resulted in the rearrangements in PE levels, which decreased after 7 days of exposure to higher temperature; these decreases correlated well with the significant loss of frost tolerance in both analyzed genotypes (Figs. 2, 4, and 6). However, only in the HFT genotype did the level of PE reach the control values, suggesting that other traits might have also influenced this phenomenon. Intriguingly, a response in PC accumulation in the HFT genotype at DA-1, which significantly affected the PE/PC ratio, could also be observed (Fig. 4). This was accompanied by the up-regulation of the gene coding phosphoethanolamine *N*-methyltransferase (*PMEAMT*) in both genotypes (Figs. 7 and 8). PMEAMT catalyzes methylation reactions of phosphobase intermediates involved in phosphocholine production, contributing to PC biosynthesis (BeGora et al. 2010). Such rearrangements may suggest a more flexible response allowing a rapid restoration of membrane composition in the HFT genotype. This could potentially explain the decrease in PE. Indeed, a rapid phospholipid remodeling during DA, including modifications in lipid class composition and degree of its unsaturation, might be a central mechanism supporting adaptation to changing environmental conditions (Hazel 1995, Uemura et al. 1995). Such dynamic adjustments could maintain membrane integrity and enable swift recovery of lipid balance following environmental fluctuations, supporting the central role of phospholipids in the DA process. Previous studies on coniferous species showed a decreased phospholipid content and a lower unsaturation level during natural spring DA (Pukacki and Kamińska-Rożek 2013). No lipidomic studies have been conducted on forage grasses during DA to date, leaving a gap in our understanding of phospholipid dynamics during this process. Additionally, genotypic differences were observed at the DA-1 time point, where lower DAG levels were detected in the LFT genotype, compared to the HFT genotype (Fig. 4), which might be related to more intensive DAG metabolism in this genotype. These differences at DA-1 might have been linked to a highly reduced expression of *NPC4* and a lower expression of *LPP2*, compared to the HFT genotype (Figs. 7 and 8).

Significant differences in PE/PC metabolism between two genotypes of *F. arundinacea* were observed in our study during RA, indicating that these genotypes used distinct strategies compared to CA. In the LFT genotype, a continuous decrease in total PC level together with a stable PE level was noticed. In contrast, the HFT genotype maintained stable PC levels while presenting a rise in total PE level and increased unsaturation level of PE, suggesting an alternative strategy of membrane remodeling (Fig. 4). Despite these contrasting trends, both genotypes exhibited an increase in PE/PC ratio during RA (Fig. 4). However, the increase in PE level, compared to DA-7 and higher PC level, compared to the LFT, in the HFT genotype at RA-21 might be responsible for its higher frost tolerance at this stage (Fig. 4). Transcriptomic regulation of crucial genes associated with lipid metabolism appeared to reflect the observed differences between the analyzed plants. In the LFT genotype, a strong down-regulation of *PMEAMT* was observed at RA-21, which might contribute to reduced PC availability by reducing phosphocholine production. In the HFT genotype, however, the *PMEAMT* down-regulation was apparently lower than that observed in the LFT, while a significant up-regulation of *PEAMT1* was observed, which could potentially help to maintain a stable level of total PC. Additionally, transcriptomic data also revealed an increase in *NPC4*, compared to DA, which might support the observed decrease in total PC in the LFT genotype (Figs. 7 and 8). Taken together, these observations suggest that the HFT genotype maintained an adaptive response during RA, potentially supported by enhanced PE biosynthesis via PCYT2. In DAG accumulation the genotypic differences were also observed at the RA-3 time point, specifically, reduced accumulation of DAG in the LFT genotype (Fig. 4). At RA-3 in both genotypes, *DGK1* expression increased, but predominantly in the LFT genotype, suggesting a potential increase in phosphorylation of DAG to PA (Tan et al. 2018), which might be related to the observed genotype differences (Figs. 7 and 8). At the RA-3 lower DAG level might have been associated with the down-regulation of *GPAT3* and *GPAT6* in the LFT genotype (Figs. 7 and 8). *NPC4* genes were upregulated in both genotypes also during RA, which may suggest that membrane lipid remodeling might provide a key source of DAG during cold treatment (Figs. 7 and 8) (Sagar and Singh 2021). These observations are also consistent with the possibility that PC contributes to DAG availability not only during CA but also during RA. Moreover, during CA, *KCS* genes, coding proteins involved in the elongation of very-long-chain fatty acids, were consistently downregulated in both genotypes (Figs. 7 and 8). This down-regulation might moderately alter the composition of membrane lipids, particularly affecting very-long-chain fatty acids (Kim et al. 2013). Our results clearly indicate the important role of PE and PC remodeling in the acquisition of frost tolerance during CA and its loss during DA process. Moreover, observed a higher level of unsaturated PE in the control conditions and during PA in the HFT genotype (Figs. 4 and 6) might be associated with genotype dependent differences in frost tolerance of analyzed plants.

### Genotypic differences in galactoglycerolipid accumulation profiles could influence the level of frost tolerance and functioning of photosynthetic apparatus under CA and RA

In our study many of the genes associated with fatty acid biosynthesis in chloroplast were upregulated under the CA including that coding 12-oxophytodienoate reductase (*OPR1_2*), which was upregulated in both genotypes but significantly higher in the HFT, acyl-[acyl-carrier-protein] desaturase (*SAD7*), which was also upregulated in both genotypes, but higher in the LFT, NADPH-dependent aldehyde reductase (*ALR*) and palmitoyl-acyl carrier protein thioesterase (*FATB*), which were upregulated only in the LFT genotype (Figs. 7 and 8). Also, transcripts of chloroplast-localized long-chain acyl-CoA synthetase 8 (LACS8) enzyme were upregulated in both genotypes, but significantly higher in the LFT during CA. This phenomenon might be associated with the activation of free fatty acids into acyl-CoA (Figs. 7 and 8). Altogether these findings suggest that fatty acid production was generally promoted during cold treatment in both genotypes, providing the source for the synthesis of chloroplast lipids including MGDG and TAG. These results, in our opinion, should have been reflected in a higher content of lipids in the LFT genotype, but in fact, in our study, the increase of TAG, MGDG, and even DGDG levels was observed only in the HFT genotype under CA (Figs. 4 and 8). Also, a stable ratio of these galactoglycerolipids was observed in the HFT, mainly due to the increased levels of MGDG 36:6 and DGDG 36:6 species, which represented the majority of identified galactoglycerolipids, and both are rich with alpha-linolenic acid residues (Burgos et al. 2011). By contrast, the LFT genotype maintained a stable total DGDG level but displayed a significant reduction in unsaturated MGDG 36:6, which negatively affected the deduced MGDG/DGDG ratio (Figs. 4 and 6). This situation might be partially a result of possible increased activity of lipoxygenase (LOX), as indicated by the significant up-regulation of *LOX2-5* genes during PA in the LFT genotype (Figs. 7 and 8). Moreover, during CA, the expression of *LOX 2-3* in the genotype HFT was significantly more downregulated, compared to the LFT, and for *LOX 4-8*, an increased expression in the LFT genotype could be observed, while the HFT genotype downregulated these genes (Figs. 7 and 8). As it was proven earlier, higher LOX activity may limit the pool of acyl-CoA available for lipid biosynthesis (Singh et al. 2022). Moreover, we could also observe up-regulation of *MGD3* gene associated with MGDG biosynthesis under CA, which remained elevated even during DA and RA in both genotypes (Figs. 7 and 8), however, its higher expression was reflected in the increase of MGDG level only in the HFT genotype during CA (Figs. 4 and 8). Transcripts of *DGD1* gene family, encoding the enzyme responsible for the conversion of MGDG to DGDG, were upregulated also in both genotypes. *DGD1_2* increased during PA and persisted in an upregulated state during CA, whereas genotype-specific patterns were observed for the remaining transcripts: *DGD1_3* increased during PA in the HFT genotype, while *DGD1_1* increased during CA in the LFT genotype. The *DGD2* gene was slightly upregulated only in the LFT genotype during CA and remained elevated afterward (Figs. 7 and 8). The stronger up-regulation of *DGD1* in the HFT genotype might be responsible for sustaining the MGDG/DGDG ratio at the control-like levels. Such a regulation might therefore support lipid homeostasis. MGDG and DGDG play distinct but complementary roles in membrane organization (Hincha et al. 1998). Both are integral components of photosynthetic systems, contributing to PSI and PSII assembly and stability as well as ensuring efficient photochemical reactions (Yoshihara and Kobayashi 2022, Zhukov and Volkov 2025). MGDG promotes an interaction between the PSII core complex and associated light-harvesting complex, facilitating PSII dimerization and energy transfer (Fujii et al. 2019). DGDG stabilizes PSI and PSII structure, including the oxygen-evolving complex of PSII and ensuring PSI subunit associations. A loss of DGDG significantly impaired photosystem stability and photochemical performance in *A. thaliana* (Guo et al. 2005). It should be highlighted that a reduction of total MGDG is widely reported as a common reaction to cold stress in many plant species, often accompanied by stable or increased DGDG levels. Previous studies have reported lipid remodeling patterns similar to those observed in the LFT genotype. In *Zea mays*, MGDG content decreased under cold stress, while DGDG either remained stable or increased (Gu et al. 2017, Gao et al. 2024). Evidence from Bermudagrass (*Cynodon spp.*) further supports this view, as cold-sensitive varieties showed a stronger decline in MGDG, whereas cold-tolerant varieties maintained higher unsaturation of MGDG, which was suggested to be important for preserving membrane functions under cold stress (Fontanier et al. 2020). Consistent with the cold tolerance strategy, the HFT genotype displayed a response characterized by an increase in MGDG unsaturation. In addition, the HFT genotype maintained the MGDG/DGDG ratio, suggesting that both parameters might contribute to response to cold stress. In general, increased MGDG content or unsaturation level may enhance membrane fluidity under cold conditions; however, excessive accumulation could destabilize the bilayer, potentially reducing membrane functionality (Uemura and Steponkus 1997, Degenkolbe et al. 2012). Therefore, MGDG/DGDG management observed in the HFT genotype might reflect a mechanism that helped to maintain membrane homeostasis during CA. This assumption could be supported by observations in *A. thaliana* (freezing-tolerant), where higher MGDG/DGDG ratio was observed, compared with *Z. mays* (freezing-sensitive), suggesting that the ability to regulate this ratio might be positively correlated with cold tolerance (Zheng et al. 2016).

The importance of maintaining a stable MGDG/DGDG ratio for the structural integrity and functional stability of thylakoid membranes under stress conditions has been emphasized in previous studies (Dörmann and Benning 2002, Makshakova et al. 2020). Distinct remodeling of MGDG/DGDG ratio under CA was associated with different photosynthetic apparatus response to low temperature in both genotypes. In our work, during CA, both genotypes achieved the similar values of φ*P*_0_ and φ*R*_0_ parameters, despite significant intergenotypic differences between them in the control conditions. It was mainly due to a decrease of their values in the HFT genotype, although this decrease was statistically not significant in the case of φ*R*_0_ (Fig. 3). That phenomenon validates our prior findings, where the accelerated acclimation of photosynthetic apparatus to low temperatures was concomitant with augmented frost tolerance (Rapacz et al. 2004, 2008a, 2008b). In our result, we could also observe that ꞵ-glucosidase (sensitive to freezing 2) (SFR2) enzyme, which catalyzes the transfer of the galactose group from MGDG to another MGDG molecule to form oligogalactolipids, could potentially contribute to a reduction in the MGDG/DGDG ratio (Roston et al. 2014). In our experiment, significant up-regulation of *SFR2* was visible only during PA, but it was reduced further. No significant changes in *SFR2* transcript levels were observed during CA, DA, and RA, likely because the plants were exposed only to low but non-freezing temperatures. Under such conditions, as reported in *A. thaliana, SFR2* transcription remains largely unchanged, and the activity of SFR2 is thought to increase primarily under freezing stress, reflecting post-translational activation (Barnes et al. 2016, Barnes et al. 2019).

During DA, a significant up-regulation of genes associated with fatty acid biosynthesis was revealed. The genes coding enoyl-[acyl-carrier-protein] reductase [NADH] 1 (*FABI1*), *OPR1_1*, and 3-oxoacyl-[acyl-carrier-protein] synthase II increased in the LFT genotype while *ALR* in the HFT. However, the expression of *FATB* and *SAD7* was reduced in both genotypes, but especially in the LFT (Figs. 7 and 8). Moreover, a down-regulation of *DGD1* gene expression in both genotypes, which was escalated in the HFT, was observed (Figs. 7 and 8). However, DA revealed almost no effect on chloroplast galactoglycerolipid remodeling in either genotype (Figs. 4 and 8). This might be partially a result of transient up-regulation of genes responsible for fatty acid degradation such as *LOX 2-3* and *HPL,* especially in the LFT genotype (Figs. 7 and 8). However, a significant decrease in MGDG unsaturation level after 7 days of de-acclimation was observed in both genotypes (Fig. 5) and it may indicate a possible adaptive shift toward a reduced membrane fluidity, which could help to stabilize thylakoid structure during DA. Differences in DGDG unsaturation levels between the genotypes might further imply that the HFT genotype was adapting better to changing environmental conditions. It cannot be excluded that this phenomenon might have been crucial in modulating membrane stability, or that the observed changes could have been responsible for a transiently preserving structural integrity under warming episodes (Stachurska and Janeczko 2024). It was observed in rapeseed that DA triggered a decrease in total MGDG and a reduction in its unsaturation level, which was interpreted as a potential mechanism to decrease membrane fluidity and to prepare chloroplasts for subsequent temperature fluctuations (Rys et al. 2024).

By contrast, our data revealed that RA resulted in a negative alteration of chloroplast membrane lipids in both genotypes, despite the up-regulation of *OPR1_2* and *SAD7* in both genotypes (Figs. 7 and 8). During this phase, the HFT genotype was characterized by stable total DGDG levels, but total MGDG levels decreased, negatively influencing the deduced MGDG/DGDG ratio starting from RA-3. While in the LFT genotype, both total pools of MGDG and DGDG decreased. These changes mostly resulted from modifications of DGDG 36:6 and MGDG 36:6 levels (Fig. 6). In this genotype, higher expression of *LOX* genes was indicated (Figs. 7 and 8). Due to galactoglycerolipids remodeling, the deduced MGDG/DGDG ratio decreased also in this genotype after 21 days of re-acclimation (Fig. 4). However, it cannot be excluded that a part of the MGDG pool was converted into DGDG, particularly in the HFT genotype, as *DGD1* was upregulated during RA. These results indicate that RA activated distinct reactions from those observed during CA. RA might trigger specific remodeling pathways affecting chloroplast membrane functionality, especially according to photosynthetic apparatus. Additionally, the genotype differences in PSII efficiency (φ*E*_0_) during RA (Fig. 3), which were mainly due to a significant decrease of that parameter in the HFT genotype, might be associated with genotype-dependent lipidome reprogramming observed in our experiment and particularly changes in the degree of galactoglycerolipid unsaturation, which might result in increased frost tolerance and winter survival (Rapacz et al. 2004, Rapacz et al. 2008a, 2008b).

### Triacylglycerol accumulation can be a crucial trait responsible for scavenging of toxic lipoxygenation products under cold stress

Low temperature stress disrupts cellular redox homeostasis, leading to excessive ROS production (including H₂O₂) and ROS-driven lipid peroxidation, particularly affecting polyunsaturated membrane lipids (Rao et al. 2025). In addition, the oxidation of polyunsaturated fatty acids catalyzed by LOX leads to the formation of oxylipins and lipid peroxides, which, due to their cytotoxicity, enhance oxidative stress, increase the levels of oxidative damage markers such as MDA and H₂O₂ (Biswas and Mano 2021, Singh et al. 2022, Knieper et al. 2023). In this context, accumulating evidence indicates that TAG might play an important protective role under stress conditions by sequestering excess free fatty acids. This phenomenon may limit substrate availability for LOX (Cagliari et al. 2011, Chapman and Ohlrogge 2012, Perlikowski et al. 2016, Perlikowski et al. 2019, Perlikowski et al. 2020, Perlikowski et al. 2022, Sánchez-Martín et al. 2018, Fan et al. 2023).

TAG biosynthesis requires the concerted activity of several enzymes, among which DGAT plays a central role by catalyzing the acylation of DAG with free fatty acids, resulting in TAG formation (Tan et al. 2018). During CA, we observed that induction of *DGAT* genes was observed in both genotypes of *F. arundinacea, DGAT1* predominantly in the LFT genotype, while *DGAT2* predominantly in the HFT genotype (Figs. 7 and 8). In *A. thaliana, DGAT1* overexpression enhanced TAG accumulation and cold tolerance, whereas *dgat1* mutants showed reduced TAG content and increased sensitivity to low temperature (Arisz et al. 2018, Tan et al. 2018). Furthermore, in *H. vulgare*, frost-tolerant cultivars exhibited up-regulation of *DGAT1* and extensive lipid remodeling under cold stress, consistent with enhanced membrane stability mechanisms in cold-adapted genotypes (Zhao et al. 2024). Additionally, we identified *PES1*, which was transcriptionally induced in both analyzed here genotypes, but significantly higher in the LFT genotype during CA. PES1 enzyme has primarily been characterized as a key enzyme involved in the biosynthesis of fatty acid phytyl esters. However, due to the presence of the acyltransferase domain, it may also contribute to TAG formation by promoting the incorporation of free fatty acids, from *de novo* synthesis or originated from MGDG degradation, into TAG (Lippold et al. 2012, Lu et al. 2020) and this observation correlates with the observed decrease in MGDG level in this genotype at CA (Figs. 4, 7, and  8). Despite the transcriptional activation of TAG-biosynthesis genes, an increased accumulation of TAG was observed only in the HFT genotype, while TAG levels decreased in the LFT genotype under CA (Fig. 4). In the LFT genotype, decreased TAG accumulation might reflect substrate competition between LOX and TAG biosynthesis, as indicated by higher accumulation of *LOX* transcripts during PA and CA, compared to the HFT genotype (Figs. 7 and 8). A higher activity of LOX in the LFT might limit the pool of acyl-CoA available for TAG biosynthesis (Singh et al. 2022). This LOX-mediated utilization of free fatty acids could generate not only a reduced substrate availability for TAG but might also correlate with the observed increase in H₂O₂ and MDA contents in this genotype (Nejadsadeghi et al. 2015, Djian et al. 2022, Qin et al. 2023) (Fig. 4). This mechanism has been further supported by studies in chickpea (*Cicer arietinum*) exposed to 4°C, where cold stress induced LOX activity alongside increased H₂O₂ and MDA levels, linking LOX activation to enhanced oxidative stress and lipid peroxidation in cold-sensitive genotypes (Javidi et al. 2022). The observed increase in H₂O₂ and MDA contents has been frequently associated with lower frost tolerance, which correlates with our results for the LFT genotype. Similar relationships between increased oxidative stress markers and reduced frost tolerance have been reported for *H. vulgare*, where frost-tolerant genotypes maintained lower levels of H₂O₂ and MDA under low-temperature conditions (Valizadeh Kamran et al. 2015). In contrast, the HFT genotype exhibited a general down-regulation of *LOX* genes during CA, lower accumulation of H₂O₂ and MDA, and a pronounced increase in TAG accumulation (Figs. 2, 4, 7, and 8). Similarly to the HFT genotype, cold stress has been associated with increased TAG accumulation in *A. thaliana* (Degenkolbe et al. 2012). In Drummond’s rockcress (*Boechera stricta*) frost-tolerant ecotypes showed higher increases in TAG levels during CA, compared with frost-sensitive ecotypes (Arisz et al. 2018). The increased TAG level observed in the HFT genotype might function as a dynamic buffer for free fatty acids released during membrane lipid remodeling. This temporary sequestration could prevent cytotoxic accumulation, restrict substrate availability for LOX, and provide a reservoir of acyl chains for membrane repair and energy needs. These mechanisms could likely support membrane integrity and enhanced frost tolerance, consistent with the proposed roles of TAG metabolism in lipid homeostasis and the adaptation to low temperature (Cagliari et al. 2011).

Beyond CA, TAG accumulation increased during DA in both genotypes, and this phenomenon was accompanied by reduced H₂O₂ levels in the LFT genotype and stabilization of MDA content in the HFT genotype (Figs. 2 and 4). After 7 days of de-acclimation, the unsaturation level of total TAG content was significantly elevated, suggesting that free fatty acids and lipid breakdown intermediates released during stress-induced membrane remodeling, particularly of unsaturated PE, were efficiently scavenged and incorporated into TAG (Fig. 5). It was previously reported that TAG synthesis might require the activity of PDAT1, which can directly transform phospholipids, usually PC, but also possibly PE, into TAG (Bates and Browse 2012). However, only slight differences in *PDAT1* transcript accumulation in analyzed genotypes during PA and CA were recognized, which were further transferred to DA without down-regulation and increased also during RA in both genotypes similarly (Fig. 7). However, the low significant expression level (FC < 2) might be correlated with an elevated amount of these transcripts already in the control conditions, which might influence the further expression (Supplementary Table S2). It cannot be excluded that this mechanism drove a conversion of PE directly into TAG during DA, or indirectly through PC, since *PMEAMT* up-regulation was observed in both genotypes at this phase (Figs. 7 and 8). The transient up-regulation of *LOX 2-3* and *HPL* genes, especially in the LFT genotype during DA (Figs. 7 and 8), might reflect slower accumulation of TAG at the beginning of DA in the LFT genotype. However, the observed increase in TAG accumulation at DA-7 potentially buffered acyl chain intermediates from lipid turnover, limiting their cytotoxic impact and contributing to H₂O₂ and MDA parameters. During DA, *DGAT1* in the HFT and *DGAT2* in the LFT genotype remained at the expression level observed during CA, but *DGAT1* in the LFT and *DGAT2* in the HFT decreased in expression slightly, and this might indicate the selective genotypic divergence in a modulation of these genes activities. Moreover, *PES1* was also highly downregulated during DA, especially in the LFT genotype, which was correlated with stable levels of MGDG and DGDG in both plants (Figs. 7 and 8).

Our data revealed that RA led to distinct genotype-dependent dynamics of TAG accumulation. In the HFT genotype, TAG levels remained relatively stable at RA-3, compared to DA-7, whereas a transient decrease was observed in the LFT genotype, which might reflect enhanced TAG turnover and mobilization of lipid intermediates during the early phase of RA (Fig. 4). After 21 days of re-acclimation, both genotypes exhibited increased accumulation of TAG, which could indicate extensive remodeling of chloroplast membranes and scavenging of lipid byproducts derived from galactoglycerolipid turnover. PES1 might play a central role in that process, since its gene was highly expressed in both plants, especially in the LFT genotype (Figs. 7 and 8), potentially transferring fatty acids obtained from galactoglycerolipid remodeling, including MGDG-derived acyl chains, supported by its chloroplast localization (Lippold et al. 2012). This was accompanied by a significant reduction in MDGD level in both plants, but still a significantly lower level was observed in the LFT genotype (Figs. 4 and 8). Additionally, we cannot exclude a potential role of DGAT1 or DGAT2 in the scavenging of lipid turnover byproducts in chloroplasts during cold treatment. DGAT1 has been suggested to associate with chloroplast membranes in higher plants (Xu et al. 2020). This is supported by Western blot detection of the protein in the chloroplast fraction and by increases in *DGAT1* expression and accumulation of TAG derived from chloroplast lipids during leaf senescence (Kaup et al. 2002). In microalgae, enzymes such as DGAT1 and DGAT2 often contain predicted chloroplast transit peptides. They are proposed to interact with plastid-adjacent membranes, although these organelles are structurally distinct from plant chloroplasts (Bagnato et al. 2017, Xin et al. 2019). In fact, in *Nannochloropsis oceanica*, two DGAT2 isoforms have been shown to localize in the chloroplast envelope using GFP fusion overlapping with chlorophyll autofluorescence, indicating plastid-associated TAG synthesis (Xin et al. 2019). While DGAT2 in higher plants is predominantly localized in the endoplasmic reticulum, observations in algae highlighted a hypothetical involvement of plastid-associated membranes in TAG synthesis. During RA, *LOX* expression decreased only in the HFT genotype, potentially reflecting lower LOX-mediated lipid oxidation under reduced oxidative stress. This was supported by a decrease in MDA levels, while accumulating TAG likely sequestered free fatty acids. Moreover, a lower DAG level in the LFT genotypes at DA-1 and RA-3 time points was accompanied by a deficit in TAG accumulation at the same time points (Fig. 4), which might limit further TAG accumulation. Taken together, these observations support the idea that TAG accumulation during CA, DA, and RA represents an integral component of stress-induced lipid remodeling and TAG is an effective metabolic scavenger of free fatty acids and lipid byproducts, sequestering potentially cytotoxic lipid species and limiting their contribution to LOX-derived oxidative damage.

## Conclusions

The CA conditions increased levels of frost tolerance in both genotypes; however, the HFT genotype consistently exhibited higher tolerance at all analyzed time points, compared with the LFT genotype. This enhanced performance was associated with coordinated remodeling of membrane lipids. In particular, the HFT maintained a balanced MGDG/DGDG ratio, which was supported by the up-regulation of *MGD1* and *DGD1* genes. In both the HFT and the LFT genotypes, an increased level of polyunsaturated PE and PC corresponded with increasing frost tolerance. However, the HFT genotype was characterized by significantly higher PE levels. The PC/PE remodeling was supported by the up-regulation of several genes involved in PE and PC synthesis, such as *PEAMT1, PCYT2, CCT2, CEPT1*, and *ETNK*, while *PEAMT1* and *PCYT2* were higher up-regulated in the HFT genotype and might contribute to its better performance. The HFT genotype also accumulated higher amounts of TAG, which might be associated with a scavenging of potentially toxic free fatty acids. Higher TAG accumulation was associated with increased transcript contents of *DGAT1, DGAT2*, and *PES1*. Despite higher up-regulation of *DGAT1* and *PES1* in the LFT genotype, a reduced TAG accumulation was observed. This trait might be related to a possible deficit of free fatty acids due to possible higher LOX activity, associated with higher transcript levels of several *LOX* genes observed in the LFT during PA and CA. This phenomenon might lead to a higher content of H_2_O_2_ and MDA in this genotype. *NPC4* and *LPP2* contributed to DAG availability for lipid synthesis, especially in the LFT genotype. Most patterns of transcript expression observed under CA were also revealed during the RA process.

Short-term DA temporarily suspended further acclimation without reducing frost tolerance. Prolonged DA led to a significant decrease in tolerance, but it was not sufficient to fully de-acclimate both genotypes. Full DA strongly affected lipid composition, especially due to decreased PE accumulation, leading to reduced frost tolerance in two analyzed genotypes. Both genotypes were also characterized by significant TAG accumulation at advanced DA, which might have been a result of scavenging byproducts from PE decomposition. Importantly, *PES1* transcript levels were downregulated during DA, indicating a reduced contribution to TAG synthesis from chloroplast-derived fatty acids, yet TAG accumulation persisted, reflecting the continued mobilization of phosphoglycerolipid intermediates probably via DGAT1, DGAT2 and PDAT1 activities. Moreover, up-regulation of *PMEAMT* under DA might be associated with a transfer of PE to PC and further supporting TAG accumulation.

During RA, strong genotypic differences in PE and PC remodeling affected frost tolerance in both plants. RA conditions allowed both genotypes to restore frost tolerance after 3 days of re-acclimation. Moreover, the HFT genotype was able to acclimate more efficiently after 21 days of re-acclimation, exceeding frost tolerance obtained during CA. The HFT genotype was characterized by an increase of polyunsaturated PE and maintained balanced PC level, what was not observed in the LFT genotype. This phenomenon was associated with a more effective restoration of frost tolerance in the HFT genotype. Moreover, both genotypes increased TAG accumulation during advanced RA which might have been associated with a disruption of chloroplast membranes due to reduced MGDG and DGDG levels, especially visible in the LFT genotype. This trait was associated with increased *PES1* expression. PES1 might regulate a transfer of galactoglycerolipids intermediates into TAG. Moreover, increased *DGAT1* and *DGAT2* expression might also be involved in a scavenging of free fatty acids and phosphoglyceolipid remodeling byproducts.

## Materials and Methods

### Plant material

The plant materials comprised two hexaploid (2*n* = 6 × = 42) *F. arundinacea* genotypes (cv. Kord), selected earlier from a broader population based on their different levels of winter hardiness under field conditions. These genotypes were germinated from single seeds, which were obtained from the collection of the Institute of Plant Genetics, Polish Academy of Sciences. The more winter-hardy genotype was further proven in the current study to also be the high frost-tolerant (HFT), and the less winter-hardy genotype—the low frost-tolerant (LFT)—at each analyzed experimental time point. Winter hardiness of the selected genotypes was evaluated during seasonal winters, based on winter bonitation level and post-winter regrowth potential (unpublished data). Experiments were performed in 2016/2017 at DANKO Plant Breeding in Szelejewo (51°17′ N, 16°22′ E), Poland.

### Experimental setup

The experiment was conducted under controlled conditions in a climate chamber (phytotron/cold room) and consisted of five stages: control, pre-hardening, CA, DA, and RA (Fig. 1). Representative plant phenotypes during the crucial steps of the experiment are shown in Supplementary Fig. S1. Plants were placed as three separate clumps per genotype in 5 dm^3^ pots filled with medium containing sand and peat (1:1). Each genotype was represented by 10 pots. Plants were first cultivated at 20°C in the greenhouse for two months to establish vegetative growth, then were transferred to the chilling chamber and subjected to a cold hardening procedure. The first stage involved the control conditions under 22°C at Day 0; the second stage was pre-hardening (PA) the plants at 12°C for 7 days, with a 10-h photoperiod and light intensity of 200 *μ*mol m^2^s^−1^ PPFD. Subsequently, the plants underwent 21 days of CA, during which the temperature was maintained at 4°C during the day and 2°C at night, with the same light parameters. After completing the CA, the DA process was initiated, starting after 21 days of acclimation. De-acclimation lasted for 7 days, during which the temperature was raised to 12°C, while light conditions remained unchanged. The next stage was RA, which began after the completion of de-acclimation and lasted for 21 days. During this phase, the plants were again exposed to conditions of 4°C during the day and 2°C at night. All the experimental conditions were maintained with constant light parameters (10-h photoperiod and 200 *μ*mol m^2^s^−1^ PPFD), with temperature as the only variable depending on the experimental stage. The control group was kept under optimal conditions at 22°C, with identical photoperiod and light intensity, as described above. The light intensity value was selected based on our previous work related to CA and frost tolerance in *Lolium-Festuca* grasses (Kosmala et al. 2006, Kosmala et al. 2009, Bocian et al. 2011, Augustyniak et al. 2018). Temperature causing 50% of electrolyte leakage (LT_50_), as well as the measurement of reactive hydrogen peroxide content (H₂O₂), the degree of lipid peroxidation (MDA), chlorophyll fluorescence and harvesting of leaf samples for the lipidomic and mRNA analyses were conducted at strictly defined time points: control (C) Day 0, the 7th day of PA, the 21st day of CA, the 1st day of de-acclimation (DA-1), the 7th day of de-acclimation (DA-7), the 3rd day of re-acclimation (RA-3), and the 21st day of re-acclimation (RA-21).

### Physiological analysis

#### LT_50_ parameter

In the present study, LT_50_ values were determined at each experimental time point. For each genotype, a second fully developed leaf was excised and segmented into 2 cm fragments. For each temperature treatment, three independent biological replicates were prepared. Sample sets were established for five target freezing temperatures: –3°C, −6°C, −9°C, −12°C, and −15°C. Each tissue segment was placed into a plastic vial containing 5 cm^3^ of deionized frozen water. This approach ensured controlled ice nucleation within the vascular tissues, simulating physiologically relevant freezing conditions encountered in plants. The vials were subsequently transferred to a programmable freezing chamber, which was initialized at 0 ± 0.5°C. Each set of leaf segments was subjected to a distinct freezing cycle corresponding to a specific target temperature. The cooling rate was maintained at 2°C per hour, and upon reaching the target temperature, samples were held isothermally for 90 min to allow for uniform freezing stress exposure. The membrane injuries were determined by measuring the electrical conductivity of tissue extract. The first conductivity measurement (*EL*_1_) was performed, after which the leaf fragments were frozen in liquid nitrogen to disrupt cell membranes and then placed back into the extract. After a 24-h incubation period, the conductivity of the extract was measured again (*EL*_2_). The LT_50_ value was calculated using formula: LT_50_ = (*EL*_1_ − *W*)/(*EL*_2_ − *W*) 100%, where (*W*) is the conductivity of distilled water (Rapacz 1999, Rapacz et al. 2010).

#### Hydrogen peroxide (H₂O₂) content

Hydrogen peroxide content was determined spectrophotometrically following the method described by Becana et al. (1986), with methodological adjustments. Fresh leaf samples (0.2 g) were homogenized in 1.5 mL of ice-cold 0.1 M potassium phosphate buffer (pH 7.8) using a TissueLyser (Qiagen) for 10 min. The homogenates were then centrifuged at 15 000 × g for 30 min at 4°C, and the resulting supernatants were collected for analysis. Each reaction mixture (1.5 mL total volume) contained 360 *μ*L of extract, 640 *μ*L of 0.1 M potassium phosphate buffer, and 500 *μ*L of freshly prepared titanium reagent. The reagent was obtained by mixing equal volumes of 0.6 mM 4-(2-pyridylazo) resorcinol and 0.6 mM potassium titanium oxalate immediately before use and stored on ice. After 10 min of incubation at room temperature, absorbance was measured at 508 nm. Hydrogen peroxide content was determined using a standard calibration curve and expressed as *μ*mol H₂O₂ g^−1^ FW. All assays were conducted in triplicate (Becana et al. 1986).

#### Lipid peroxidation—level of thiobarbituric acid reactive substances

The level of lipid peroxidation was estimated as the amount of thiobarbituric acid reactive substances (TBARS) according to the method of Heath and Packer (1968). Fresh leaf samples (0.2 g) were homogenized in 1.4 mL of 25% thiobarbituric acid (TBA) in 10% trichloroacetic acid (TCA) and further homogenized using a TissueLyser for 10 min. Three biological replicates were performed for each genotype; one blank sample containing 0.2 g of fresh tissue with only 10% TCA was included. The samples were centrifuged at 1000× g for 10 min at 4°C. The supernatant was transferred to a new tube. Absorbance was measured at wavelengths of 535 and 600 nm. The amount of TBARS was expressed as malondialdehyde (MDA) equivalents, where MDA represents a major end product of lipid peroxidation and serves as an indicator of oxidative damage in plant tissues (Heath and Packer 1968).

#### Chlorophyll fluorescence

Fluorescence measurements were performed at plant growth temperature (variable during the experiment) in the second hour of the day, in the central part of leaves adapted to darkness for 20 min using a factory clip. Chlorophyll fluorescence transient measurements were performed using a Handy PEA fluorometer (Hansatech, Kings Lynn, UK), with a saturating light pulse of 2500 *μ*mol·m^−2^·s^−1^, a pulse duration of 0.5 s, and a constant amplification factor (1×). The saturation efficiency of this signal was checked each time by analyzing the fluorescence induction curve (Handy PEA software, v. 1.30, Hansatech). Measurements in which the signal was not saturated (*F_m_* not reached) were rejected. Individual parameters were calculated according to Strasser et al. (2004) and Strasser and Tsimilli-Michael (2001).

#### Extraction of lipids

For each time point, three individual leaf samples, homogenized into fine powder, were prepared. Then for each sample, 20 mg of tissue was transferred into 2 mL Eppendorf tubes for liquid extraction. The extraction procedure was based on the previously described method (Giavalisco et al. 2011, Perlikowski et al. 2016, 2022). Each sample was combined with 1 mL of cold (−15°C) extraction solvent composed of methyl-tert-butyl ether, methanol, and water in a 3:1:1 ratio (v/v/v). The mixture was gently agitated on an orbital shaker at 4°C for 10 min and then subjected to 10 min of sonication. Subsequently, 500 *μ*L of a water:methanol solution [3:1, ultra-performance liquid chromatography (UPLC) grade] was added, and the sample was thoroughly mixed. After centrifuging at 20 000 × g for 5 min at 4°C, the upper organic phase was carefully collected, dried under vacuum, and stored at −80°C until further processing.

### UPLC-mass spectrometry of polar lipids

Frozen dried lipophilic phase samples stored at −80°C were resuspended in a 7:3 (v/v) mixture of acetonitrile and isopropanol by adding 300 *μ*L of the solvent to each sample. After gentle vortexing and brief incubation at room temperature, the samples were sonicated for 5 min to enhance lipid extraction. Centrifugation was then performed at 13 000 g for 3 min, and 100 *μ*L of the resulting supernatant was transferred to analytical vials for further analysis. Lipid profiling was conducted at the Max Planck Institute of Molecular Plant Physiology in Potsdam, Germany, using a Waters UPLC system (Manchester, UK) equipped with a C8 reversed-phase column (100 × 2.1 mm, 1.7 *μ*m particle size). Separation was carried out using a 22-min linear gradient at a flow rate of 400 *μ*L/min, employing two mobile phases: phase A (water with 1% 1 M ammonium acetate and 0.1% acetic acid) and phase B (acetonitrile:isopropanol, 7:3, v/v, with the same additives). A 2 *μ*L sample volume was injected for each run. Mass spectrometric detection was performed using an Exactive mass spectrometer (Thermo-Fisher Scientific, USA) operating in alternating full-scan and all-ion fragmentation modes. The scan range was set from *m/z* 150 to 1500 with a resolution of 10 000 and a scan rate of 10 scans per second. The capillary voltage was set to 3 kV, with the capillary and ESI source temperatures maintained at 150°C and 350°C, respectively. Sheath gas and auxiliary gas flow rates were set to 60 and 35 arbitrary units (Giavalisco et al. 2011). The output from UPLC-MS (chromatographs and mass spectra) was processed in the program Refiner MS® software to detect peaks and remove fragmentation information, isotopic peaks, and chemical noise. Obtained results were assigned to lipid species based on retention time and *m/z* values (Bromke et al. 2015) (Supplementary Table S1). Detailed information on the data processing, lipid identification, and DBI calculation formula is provided in the publication of Perlikowski et al. (2016).

### Transcriptome analysis

Total RNA was extracted from the samples (three biological replicates for time-point) using the RNeasy Mini Kit (QIAGEN, Germany), in accordance with the manufacturer’s protocol, and subsequently treated with RNase-Free DNase Set (QIAGEN) to remove any remaining genomic DNA. RNA quality and integrity were assessed in a two-step process. An initial evaluation was carried out using the Agilent 4200 TapeStation system with the RNA ScreenTape assay (concentration range: 25–500 ng/*μ*L; fragment size: 50–6000 nucleotides). To confirm RNA integrity (RIN), a secondary quality assessment was performed using the Agilent 2100 Bioanalyzer. The minimal threshold of RIN value acceptance was 7. RNA sequencing was conducted by Macrogen Europe (headquartered in Seoul, South Korea) using the TruSeq Stranded mRNA Library Prep Kit (Illumina) and an Illumina sequencing platform. Sequencing was performed in a paired-end mode (2 × 151 bp). The analysis was based on the reference genome of *L. perenne* MPB_Lper_Kyuss_1697 (GCF_019359855.1). Raw sequences are available in NCBI BioProject ID: PRJNA1442041. *Lolium* and *Festuca* are genetically closely related species, and it is possible to create intergeneric hybrids within the *Lolium/Festuca* complex. The selected genome of *L. perenne* is complete and annotated, compared to other genomes available for *Festuca* species. After sequencing, trimmed reads were mapped to the reference genome (MPB_Lper_Kyuss_1697) using HISAT2, and the overall mapping ratio was evaluated. Transcript assembly was then performed for each sample using StringTie. Expression profiles for each gene and transcript were quantified as read counts, fragments per kilobase per million mapped reads, and transcripts per kilobase million. DEGs were identified across 12 pairwise comparisons using DESeq2 (Love et al. 2014), applying thresholds of |fold change| ≥ 2 and raw *P*-value < 0.05.

### Statistical analysis

The DBI was used to estimate the overall degree of lipid unsaturation. The index was calculated from log-transformed lipid intensities using a formula originally described by Orlova et al. (2003) and modified by Perlikowski et al. (2016):


$$\mathrm{DBI}=\frac{\left(\begin{array}{@{}c@{}} {\varSigma}_{i\in{I}_1}i+2{\varSigma}_{i\in{I}_2}i+3{\varSigma}_{i\in{I}_3}i+4{\varSigma}_{i\in{I}_4}i+5{\varSigma}_{i\in{I}_5}i\ \\{}+6{\varSigma}_{i\in{I}_6}i+7{\varSigma}_{i\in{I}_7}i+8{\varSigma}_{i\in{I}_8}i+9{\varSigma}_{i\in{I}_9}i\ \end{array}\right)}{\left( 100+{\varSigma}_{i\in{I}_0}i \right)}$$

Here*, Ij* represents the set of mean log_2_-transformed intensities for lipid species with *j* double bonds, where *j* = 0,1,…,9.

Statistical analyses for physiological parameters and lipid profiles were conducted using Statistica version 13.1 (StatSoft Inc., Tulsa, OK, USA). A two-way analysis of variance was applied to examine the main effects and interactions between genotype and time point. Post hoc comparisons were performed using Fisher’s least significant difference (LSD) test, with statistical significance set at *P* < 0.05. Groups not differing significantly were denoted by the same letter in graphical data presentations.

## Supplementary Material

Supplementary_materials_pcag073

## Data Availability

The transcriptomic data underlying this article are available in the GenBank Nucleotide Database at https://www.ncbi.nlm.nih.gov and can be accessed with BioProject ID: PRJNA1442041. The other data underlying this article are available in supplementary files. Further inquiries can be directed to the corresponding author.
