# Supplementary material for: Remodeling of leaf lipidome during cold acclimation, de-acclimation, and re-acclimation influences frost tolerance of Festuca arundinacea
Source: Plant Cell Physiol. 2026 May 29;67(9):1533–56. doi: 10.1093/pcp/pcag073 (PMC13621223; doi:10.1093/pcp/pcag073)
Supplement: Supplementary file 1 [file supplementary_materials_pcag073.zip › Supplementary material.docx]

**Supplementary Figure S1**. Representative images of *F. arundinacea* genotypes during the experiment. The LFT (low frost tolerant) plants are shown on the left and the HFT (high frost tolerant) plants on the right. Images A–B: control (C); C–D: cold acclimation (CA); E–F: the seventh day of de-acclimation (DA-7); G–H: the twenty-first day of re-acclimation (RA-21); I–J: control plants after fifty-six days of the experiment.

**Supplementary Figure S2.** Number of up- and down-regulated genes (FC ≥ 2, p < 0.05) in the LFT (low frost tolerant) and the HFT (high frost tolerant) genotypes of *F. arundinacea* at seven time points of the experiment: the control conditions (C), pre-acclimation (PA), cold acclimation (CA), the first day of de-acclimation (DA-1), the seventh day of de-acclimation (DA-7), the third day of re-acclimation (RA-3) and the 21st day of re-acclimation (RA-21). Yellow bars indicate upregulated and blue bars downregulated transcripts.

**Supplementary Figure S3.** Enrichment map of lipid metabolic pathways based on the abundance of transcripts of the LFT and the HFT genotypes of *F. arundinacea* during pre-acclimation (PA), cold acclimation (CA), the first day of de-acclimation (DA-1), the seventh day of de-acclimation (DA-7), the third day of re-acclimation (RA-3) and the 21st day of re-acclimation (RA-21), compared to control (C). Color scale indicates enrichment map test p-values: dark blue – p ≤ 0.001, blue – p ≤ 0.01, light blue – p ≤ 0.05, white – p > 0.05.

**Supplementary Figure S4.** Correlation analysis between samples of the LFT and the HFT genotypes of *F. arundinacea* at seven experimental time points based on Pearson’s correlation coefficient calculated from rlog-transformed expression values. The coefficient (r) ranges from –1 to 1, with values closer to 1 indicating higher similarity between samples. The heatmap illustrates the correlation matrix for all analyzed samples.

**Supplementary Table T1.** Normalized mass spectral intensity data and database annotations for the identified lipid species, together with ANOVA results. Data are shown for seven experimental time points: control (C), pre-acclimation (PA), cold acclimation (CA), first day of de-acclimation (DA-1), seventh day of de-acclimation (DA-7), third day of re-acclimation (RA-3), and 21st day of re-acclimation (RA-21), measured in *Festuca arundinacea* for the LFT (low frost tolerant) and the HFT (high frost tolerant) genotypes, based on log2-transformed normalized mass spectral peak intensities.

**Supplementary Table T2.** Differentially expressed genes (DEGs) in the LFT and the HFT genotypes of *Festuca arundinacea* involved in the biosynthesis and degradation of major lipid classes. DEGs with |log₂FC| ≥ 3 are presented here, covering pathways for PC, PE, MGDG, DGDG, TAG, and DAG. Fold-change values represent transcript accumulation at seven experimental time points relative to control (C): pre-acclimation (PA), cold acclimation (CA), first day of de-acclimation (DA-1), seventh day of de-acclimation (DA-7), third day of re-acclimation (RA-3), and 21st day of re-acclimation (RA-21). For each gene, the table provides gene and protein identifiers, gene symbol, functional annotation, log₂ fold-change values, baseMean expression, log₂ fold-change standard error (lfcSE), test statistics, raw p-values, and FDR-adjusted p-values.
